# Blockade of the BAK Hydrophobic Groove by Inhibitory Phosphorylation Regulates Commitment to Apoptosis

**DOI:** 10.1371/journal.pone.0049601

**Published:** 2012-11-26

**Authors:** Abul Azad, Joanna Fox, Sabrina Leverrier, Alan Storey

**Affiliations:** Department of Oncology, Weatherall Institute of Molecular Medicine, University of Oxford, John Radcliffe Hospital, Oxford, United Kingdom; Karolinska Institutet, Sweden

## Abstract

The BCL-2 family protein BAK is a key regulator of mitochondrial apoptosis. BAK activation first involves N-terminal conformational changes that lead to the transient exposure of the BAK BH3 domain that then inserts into a hydrophobic groove on another BAK molecule to form symmetric dimers. We showed recently that post-translational modifications are important in the regulation of BAK conformational change and multimerization, with dephosphorylation at tyrosine 108 constituting an initial step in the BAK activation process. We now show that dephosphorylation of serine 117 (S117), located in the BAK hydrophobic groove, is also critical for BAK activation to proceed to completion. Phosphorylation of BAK at S117 has two important regulatory functions: first, it occludes the binding of BH3-containing peptides that bind to BAK causing activation and cytochrome *c* release from mitochondria; second, it prevents BAK-BH3:BAK-Groove interactions that nucleate dimer formation for subsequent multimerization. Hence, BH3-mediated BAK conformational change and subsequent BAK multimerization for cytochrome *c* release and cell death is intimately linked to, and dependent on, dephosphorylation at S117. Our study reveals important novel mechanistic and structural insights into the temporal sequence of events governing the process of BAK activation in commitment to cell death and how they are regulated.

## Introduction

Members of the BCL-2 family of proteins are the major regulators of the mitochondrial (or intrinsic) apoptotic pathway whose activity is exerted through a network of intermolecular interactions with other family members. BCL-2 proteins can have either anti- or pro-apoptotic functions, and are intimately involved in the permeabilization of the mitochondrial outer membrane (MOM) that permits the release of apoptotic factors such as cytochrome *c* in response to developmental cues or cytotoxic insults, including DNA damage [1], [2]. A pivotal step in MOM permeabilization is the oligomerization of the pro-apoptotic proteins BAK or BAX, whose activation involves a number of conformational changes including the exposure of epitopes near the N-terminus [3] followed by homo-oligomerization to form pores in the outer mitochondrial membrane [4], [5], [6]. Binding of BH3-only BCL-2-family proteins result in N-terminal conformational changes in the early stages of BAK/BAX activation. This was thought to occur via one of two mechanisms that may involve either an indirect activation – where BH3-only proteins bind to and neutralize anti-apoptotic BCL-2 family members that constitutively bind to BAK/BAX in healthy cells, or by the direct binding of ‘activator’ BH3-only proteins such as BID to BAK/BAX, reviewed in [7]. Different modes of action of BCL-2-like proteins has been proposed in order to explain differences between the sequestration and direct activation models [8]. In addition, p53 may act in an analogous way to activator BH3-only proteins by binding directly to either BAK or BAX [9], [10], but this occurs at a site on BAK distinct from that involved in binding of BH3-only proteins [11].

Current evidence suggests that BAK and BAX are activated in a step-wise manner both eventually forming multimers that are believed to form pores in the outer mitochondrial membrane to release cytochrome *c* but may be differentially engaged by different apoptotic stimuli [12], [13]. In healthy cells BAX is found as an inactive cytosolic form that was shown recently to be targeted for mitochondrial translocation and homo-oligomerization by the transient binding of activator BH3-only proteins to a site near the N-terminus [14], causing intra-molecular rearrangements leading to membrane insertion by release of the α9 helix that otherwise would occlude the hydrophobic binding pocket [15]. Subsequent rearrangement of BAX/BH3 interactions to then involve the BH1 and BH3 domains further contributed towards BAX oligomerization [14]. In contrast, BAK does not appear to possess a binding site for BH3 proteins near the N-terminus, and is normally located in the outer mitochondrial membrane. As BAK does not require membrane insertion as part of the activation process and the BH3-binding groove is never occupied by the BAK α9 helix, it is likely that an alternative mechanism prevents aberrant binding of BH3 proteins to the exposed surface groove.

A model has been proposed regarding the mechanism of BAK oligomerization, whereby BAK first exposes its BH3 domain that then inserts into a hydrophobic surface groove on a second BAK molecule – termed the BH3:Groove model [16]. This resulted in the formation of symmetric dimers that could then go on to assemble into higher order structures capable of permeabilizing the mitochondrial membrane using an interface between α6:α 6 helices [17]. Further evidence from EPR studies indicated that BAK underwent large conformational changes upon multimerization and supported this model of BAK dimer and mulitmer formation [18]. In this model, the interaction of BH3-only proteins with the BAK groove must necessarily be transient if subsequent stable BAK-BH3:BAK-Groove interactions are required for dimer formation.

Our approach of studying post-translational modifications has established a key role for dephosphorylation in BAK activation and shed light on signalling pathways that impinge upon BAK activation. We found that in undamaged, otherwise healthy cells, the majority of BAK molecules appear to have a pI of ∼3.4 by 2D gel analysis suggesting that the phosphor-equilibrium is shifted in favour of the phosphorylated state. Further, dephosphorylation of BAK at Y108 constitutes the initial step in the activation process, an obligatory step required for BAK to undergo all subsequent conformational changes that lead to cell killing [19]. The significance of the many phosphorylations in terms of BAK function remains poorly understood. Our studies now reveal that in order for BAK to multimerize it must also undergo a second dephosphorylation event mediated by PP2A. This occurs at a serine residue located on its BH1 helix that forms part of the hydrophobic BH3 binding surface. Removal of this inhibitory phosphate moiety permits the interaction of BAK with BH3 sequence-containing peptides from proteins that cause N-terminal conformational change, and also relieves the restriction of BAK to the monomeric inactive form by permitting BH3:Groove interactions to occur that are critical for dimerization followed by multimerization and cytochrome *c* release.

## Results

### Apoptotic signals require PP2A activity to multimerize BAK

We previously established that changes in BAK phosphorylation at Ser/Thr and Tyr following apoptotic stimuli were involved in the activation process [19]. We found that dephosphorylation at Y108 constituted the initial step in BAK activation [19], [20], hence we hypothesized that dephosphorylation at Ser and/or Thr residues may be important for subsequent steps in BAK activation. We used HCT116 cells that lack both BAX and BAK expression and reconstituted them with wild-type BAK (which we henceforth term HCT116-BAK cells) to test whether inhibition of Ser/Thr phosphatase activity impacted on BAK activation. Since BAK activation is thought to involve two major steps, N-terminal conformational change followed by multimerization, we first used a FACS based assay to determine whether BAK could undergo N-conformational change when cells were exposed to inhibitors that block the major Ser/Thr phosphatase pathways. Low doses of okadaic acid (OA) were used to inhibit PP2A, Cyclosporin A (CyA) to inhibit PP2B or PPI-2 to inhibit PP1. We found that none of the phosphatase inhibitors tested had any impact on the ability of BAK to undergo N-terminal conformational change following UV damage (Figure S1).

**Figure 1:**
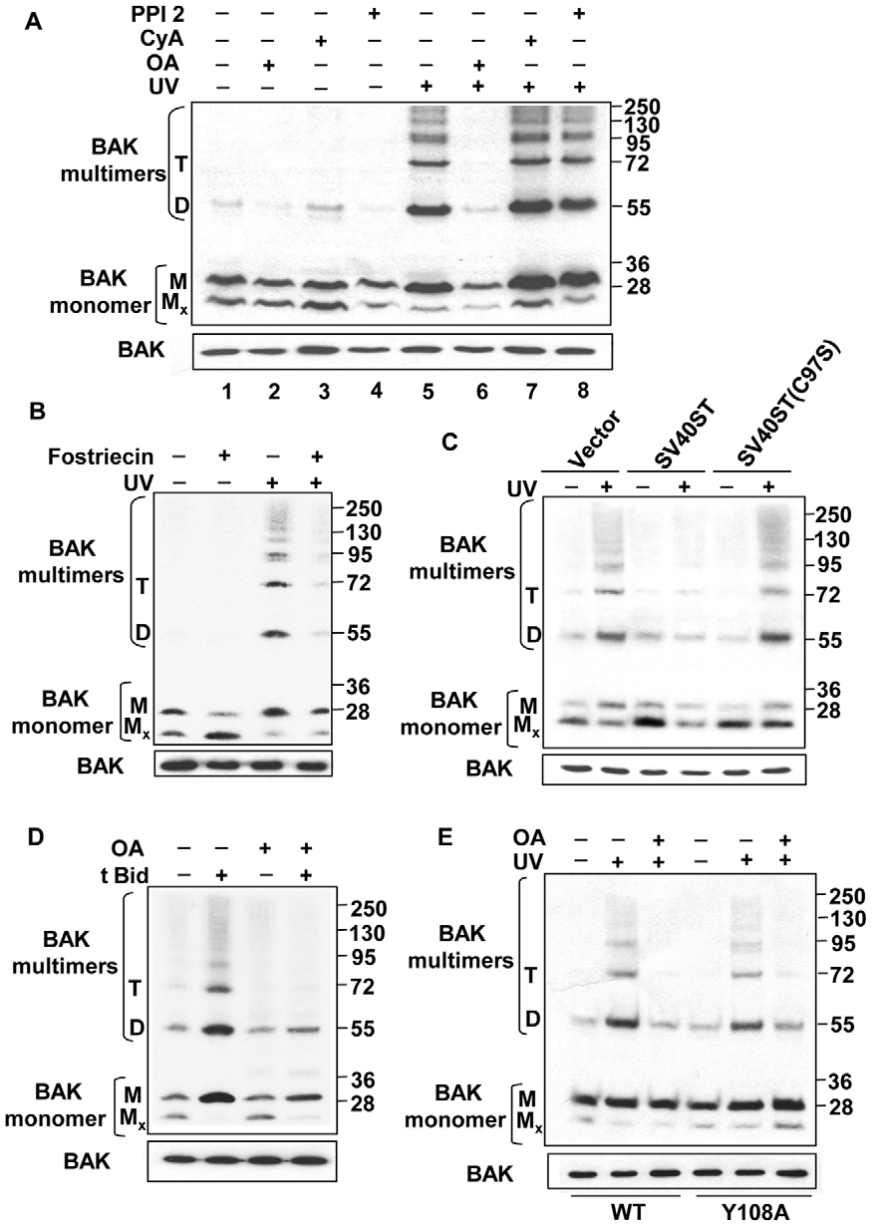
PP2A is required for BAK multimerization after DNA damage. BAK multimerization following DNA damage was assessed in HCT116-BAK cells following DNA damage. In each case a representative blot of at least 3 biological replicates is shown. In all cross-linking experiments 5% input levels of mitochondrial preparations were used to ensure equal BAK loading. (**A**) HCT116-BAK cells were pre-treated as indicated with inhibitors to major serine/threonine phosphatases for 30 mins followed by treatment with UV radiation, OA (lane 2 and 6), CyA (lane 3 and 7) and PPI2 (lanes 4 and 8), or left untreated (lane 1). 8 hrs post-irradiation mitochondrial enriched sub-cellular fractions were prepared and exposed to cross-linking agent BMH. In all multimerization assays proteins were then separated by SDS-PAGE gel electrophoresis and BAK detected by immunoblotting using the rabbit monoclonal antibody Y164. Non cross-linked BAK runs as a monomer (M) and also as intra-molecularly linked monomer (Mx). BAK dimers (D) and trimers (T) and higher molecular weight species are indicated. Only pre-treatment with the PP2A inhibitor OA resulted in the failure to detect BAK multimers. (**B**) Cells were seeded and irradiated as in (A), except that they were pre-treated with fostriecin, a potent inhibitor of PP2A activity. Mitochondria were isolated from HCT116-BAK cells ± UV damage, and protein cross-linking was performed with BMH, before BAK multimers were resolved and detected. (**C**) HCT116-BAK cells were transfected with either empty vector, or constructs expressing wild-type Mitochondria isolated from cells expressing wild-type SV40 ST or a SV40 ST (C97S) mutant that cannot bind to and inhibit PP2A. Cells were then irradiated with UV or left undamaged. Mitochondrial extracts were prepared, treated with BMH and BAK multimers resolved and detected as above. (**D**) Mitochondrial preparations treated ± OA were isolated from HCT116-BAK cells. Equal amounts of extract were incubated with purified recombinant tBid protein (5 ng/μl), then cross-linked with BMH before BAK multimers were resolved and detected. (**E**) HCT116-BAK or HCT116-BAKY108A cells were treated ± UV and ±OA. Mitochondrial extracts were prepared 8 hrs post-UV as before. OA inhibited multimerization of both wild-type and Y108A BAK indicating that PP2A dephosphorylation of BAK occurs after dephosphorylation of Y108.

We next investigated whether the phosphatase inhibitors had any effect on BAK multimerization. Mitochondrial-enriched sub-cellular fractions were prepared from cells pre-treated with OA, CyA or PPI-2, and then exposed to the sulfhydryl-reactive cross-linker BMH to crosslink BAK proteins [21]. Following UV treatment BAK dimers and higher-order complexes could be readily detected, however pre-treatment with OA, but not the other inhibitors tested, resulted in a dramatic inhibition of the ability of BAK to form multimers following DNA damage (Figure 1A). We noticed however that treatment of the cells with the BMH cross-linker could, on occasion, result in reduced detection of monomeric BAK, even though the total protein input levels were equivalent.

Although BMH has been widely used to investigate BAK multimerization, we found variability in the detection of monomeric BAK following BMH treatment that was not restricted to particular samples across repeated experiments, but instead appeared to occur in an unpredictable manner. Further, BMH also appeared to reduce band intensities of cross-linked BAK on western blots. To overcome some of these difficulties, we used a different cross-linker, BMOE, that cross-links only the dimer form of BAK rather than the higher order complexes seen with BMH [17]. Using different aliquots of the same extracts treated with either BMH or BMOE allowed direct comparison of the different reagents, confirmed that OA treatment inhibited BAK dimerization (for BMOE) or dimer and subsequent multimer formation (for BMH) following UV treatment (Figure S2A). Furthermore, OA was able to inhibit BAK multimerization in response to treatment with two other DNA damaging agents, etoposide and camptothecin (Cpt), indicating that PP2A activity was likely to be a general requirement for BAK multimerization following different apoptotic stimuli (Figure S2B, C).

To further confirm the involvement of PP2A in BAK activation, cells were pre-treated prior to damage with fostriecin, another potent and specific PP2A inhibitor. We found that fostriecin was also able to effectively block BAK multimerization following UV damage (Figure 1B). In addition to these inhibitor-based assays, we also used the SV40 small t (ST) antigen to inhibit PP2A activity [22], [23]. Expression of wild-type ST in HCT116-BAK cells (Figure S3A) reduced greatly BAK oligomerization following UV damage as evidenced by the amount of higher order oligomers formed. In contrast the C97S ST mutant, that has been shown cannot associate with PP2A, had no discernable effect on the ability of BAK to form higher order multimers following UV damage (Figures 1C). In addition to cell-based assays, BAK multimerization has been extensively studies in simpler *in vitro* models using isolated mitochondria, where addition of the truncated caspase-cleaved form of Bid (tBid) induces BAK multimerization [21], [24]. When purified tBid was added to mitochondria isolated from healthy HCT116-BAK cells that were not subject to any phosphatase inhibition, BAK multimerization was readily observed as expected, in-line with previous studies. However, mitochondria isolated from cells treated with OA were refractive to BAK multimerization induced by the addition of tBid (Figure 1D). Our previous work had demonstrated that dephosphorylation of Y108 was the initial event in BAK activation, and in line with this, we found that OA was able to inhibit multimerization of the BAK Y108A mutant following DNA damage (Figure 1E).

Overall, these findings using multiple independent approaches indicate a requirement for PP2A activity in mediating BAK activation. Importantly, as PP2A inhibition impaired multimerization but not N-terminal conformational change of the BAK Y108A mutant, we conclude that PP2A is required at later point in the BAK activation process following initial dephosphorylation of Y108. Thus a temporal sequence of dephosphorylation events is required for BAK activation to proceed to completion.

### PP2A B56α is involved in BAK-mediated cytochrome *c* release and caspase 3 activation

PP2A is the major ser/thr phosphatase in mammalian cells and plays a central role in regulating multiple intracellular signalling pathways important in oncogenesis. Mounting evidence indicates that PP2A acts a tumour suppressor [25]. PP2A activity can be down-regulated by several mechanisms including mutation of specific subunits, over-expression of intracellular inhibitors such as CIP2A [26] or interaction with viral oncoproteins such as SV40 small t antigen (ST). In immortalized cells specific silencing of the PP2A B56γ3 regulatory subunit was found to mimic ST in cell transformation, while a role for the B56α subunit was reported to be involved in PP2A regulation of BCL-2 phosphorylation in response to ceramide [27].

**Figure 2:**
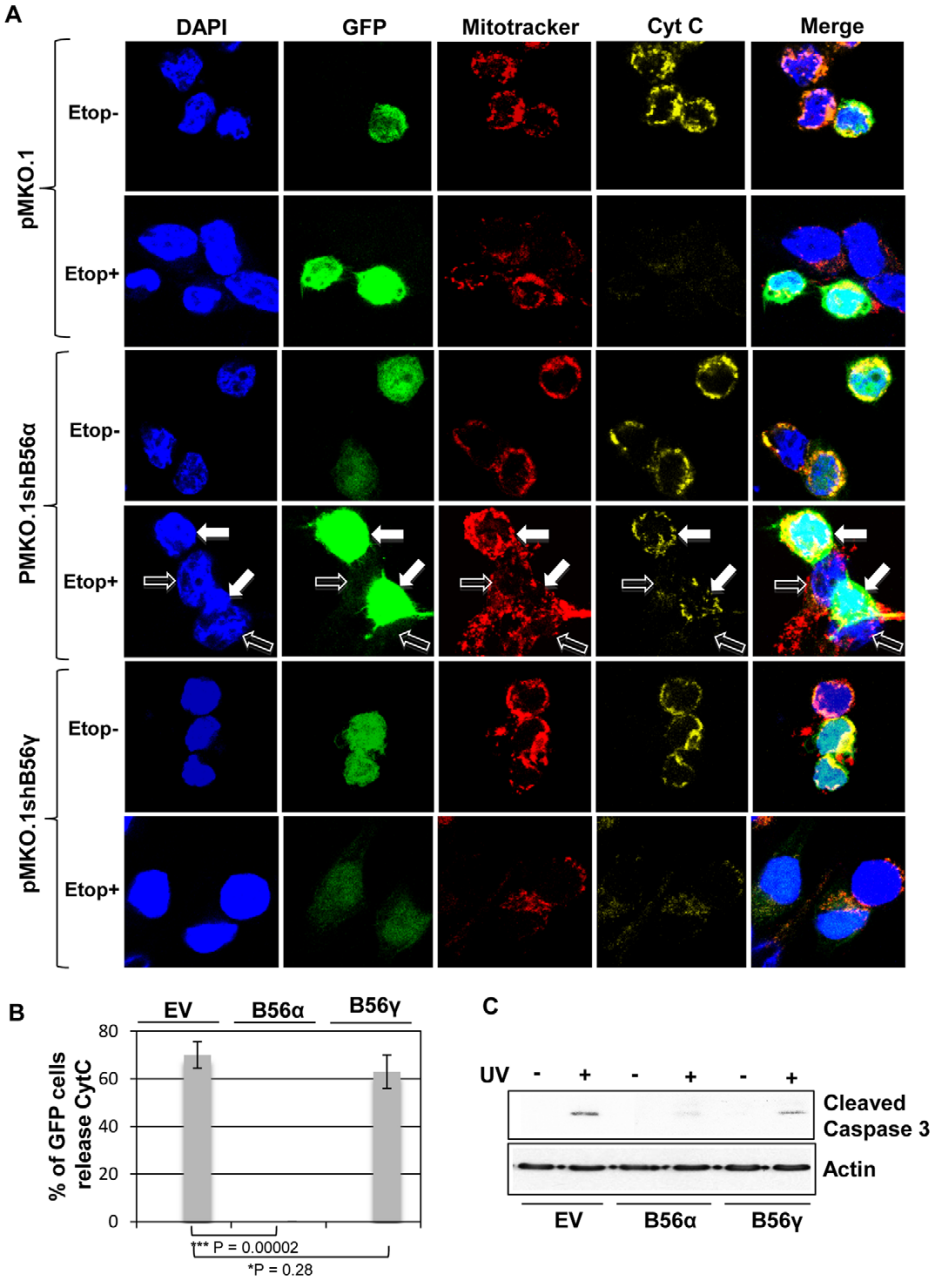
Cytochrome *c* release in HCT116-BAK cells following DNA damage requires PP2A-B56α. (**A**) HCT116-BAK cells were transfected with empty vector pMKO.1, (top panels), pMKO.1-B56α (middle panels) or pMKO.1-B56γ shRNA constructs (bottom panels). Transfection efficiency was monitored by GFP expression. After 48 hrs, cells were treated with etoposide for 24 hrs, fixed and processed for cytochrome c staining. Mitochondria was localized by staining with Mitotracker deep red FM, cytochrome c by immunofluorescence and DNA by DAPI. Representative images of three independent repeats were captured by confocal microscopy. Etoposide treatment of empty vector or B56γ-silenced cells resulted in decreased detection of cytochrome *c* localized to mitochondria. Silencing of the PP2A B56α subunit resulted in failure of cells to release cytochrome *c* following etoposide treatment with cytochrome c showing strong staining that co-localized to mitochondria. Solid arrow: cell transfected with pMKO.1-B56α (GFP positive) showing retention of cytochrome *c*, open arrow: a non-transfected cell (GFP negative) that released cytochrome *c*. (**B**) Quantification of the number of cells with cytochrome *c* release following etoposide treatment in (A). Multiple images were captured from randomly selected field of view. At least 100 cells were examined from the three independent experiments and the percentage of cells with cytochrome *c* release was determined. Error bars ±S.E.M. (**C**) Total lysates were prepared from HCT116-BAK cells transfected with pMKO.1 empty vector (EV), B56α or B56γ shRNA ± UV treatment. Following fractionation, active caspase 3 was detected by immunoblotting. Silencing of B56α but not B56γ resulted in failure to activate caspase 3. Actin was used as a loading control.

To examine the effects of PP2A on BAK, we used shRNA silencing to decrease expression of either the B56α or B56γsubunits, as evidenced by decreased levels of the proteins detected by western blotting of whole cell extracts (Figure S3B), to determine which was most important for cytochrome *c* release from mitochondria and subsequent downstream activation of caspase 3. The co-expression of GFP from the shRNA pMKO.1 vector [28] was used to monitor transfected cells. Following DNA damage, a decrease in mitochondrial cytochrome *c* staining was evident in control cells. However, silencing of B56α was found to inhibit cytochrome *c* release from mitochondria following DNA damage in transfected cells that were GFP positive. shRNA inhibition of B56γ expression resulted in only a modest decrease in total protein levels as judged by western blot, but this had little or no effect on cytochrome *c* release (Figure 2A, B), suggesting that the B56α subunit was most likely involved in mediating BAK activation and cytochrome *c* release. Exposure of HCT116*^bax−/−bak−/−^* cells, or cells expressing a BAK mutant that had a negatively charged residue replacing S117 (S117E), failed to release cytochrome *c* after etoposide treatment (Figure S4). Release of cytochrome *c* following DNA damage was accompanied with a concomitant activation of caspase 3 (Figure 2C). Taken together, our results now provide evidence that the PP2A B56α subunit is involved in and is required for BAK activation to proceed to completion, indicating that PP2A acts not only on anti-apoptotic BCL-2 proteins but also directly impinges upon pro-apoptotic cell death effectors.

### BAK dephosphorylation at the BH3:Groove interface is an essential step in activation

**Figure 3:**
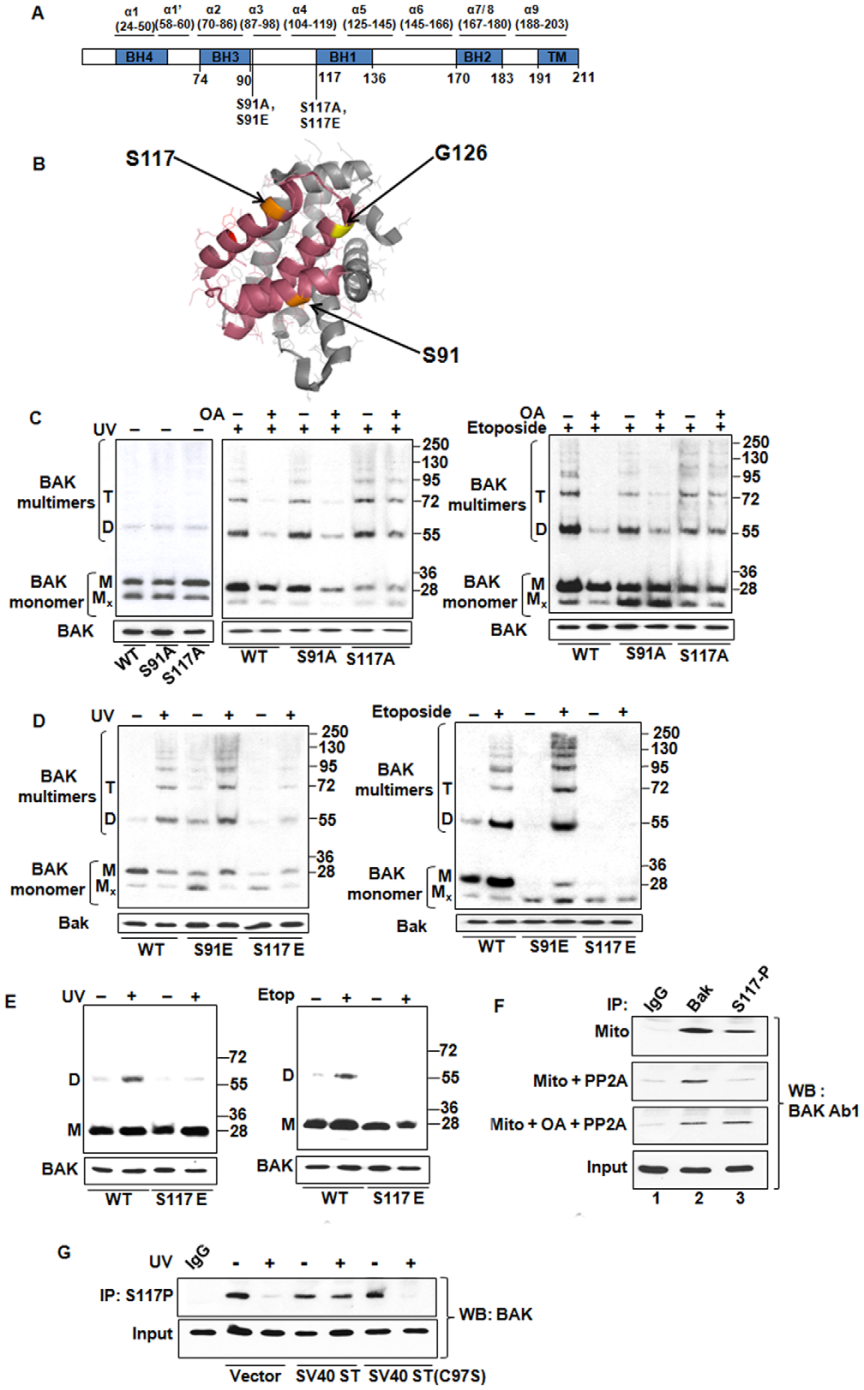
S117 dephosphorylation is required for BAK multimerization. (**A**) Schematic representation of BAK showing the relative location of the BH domains, TM domain and α-helical regions. Location of mutations of S91 and S117 are indicated. (**B**) PyMol structural representation of BAK (2IMS) with the location of S91 and S117 indicated (orange). S117 is facing the hydrophobic groove (coloured magenta) whereas S91 is outside the groove. Location of G126 is shown, mutation of G126E in the hydrophobic groove (yellow) inhibits BAK activation. Dephosphorylation of Y108 (red) has been shown previously to be the initial step of BAK activation. (**C**) BAK multimerization assay using mitochondrial extracts prepared from cells expressing HCT116-BAK, -BAK S91A or -BAK S117A mutants. Mitochondria were isolated from cells either untreated (left panel) or UV treated and also from cells treated with OA for 30 min prior treated with UV (middle panel). In the right panel cells were damage with etoposide. Mitochondrial proteins were cross-linked with BMH, resolved and detected as before. Representative blot of at least three independent repeats with input BAK levels is shown. (**D**) BAK multimerization assay using mitochondrial extracts prepared from cells expressing HCT116-BAK, -S91E or -S117E mutants. Mitochondria isolated from cells either treated ± UV (left panel). On the right panel untreated and etoposide treated cells were processed for multimerization. Representative blot of at least three independent repeats with input BAK levels is shown. Note that exposure of the S117E mutant to BMH interferes with detection of BAK complexes. (**E**) BAK multimerization assays were performed as above on the same mitochondrial enriched extracts used in (D) above except that an alternative cross-linker (BMOE) was used in place of BMH. Note that BMOE detects only monomeric and dimeric forms of BAK. (**F**) Purified active PP2A directly dephosphorylated BAK at S117. Mitochondrial extracts from HCT116-BAK cells were prepared as before and were divided into 3 equal fractions. One fraction was left untreated (top panel), another incubated with purified PP2A (middle panel) and a third was treated with OA for 30 min before PP2A incubation (bottom panel). The level of phospho S117 BAK in each extract was determined using the anti-pS117 serum. Immunoprecipitations were performed with IgG (lane 1), rabbit anti-BAK Y164 (lane 2) and anti phospho-S117 (lane 3) using extracts from all 3 fractions. Proteins present in IP reactions were detected by immunoblotting using a murine BAK monoclonal antibody (Ab1). While the anti-BAK Y164 serum was able to precipitate BAK under all conditions, PP2A treatment abolished the ability of the anti-pS117 serum to immunoprecipitate BAK, however pre-treatment with OA restored the ability of the anti-pS117 serum to IP BAK. (**G**) Inhibition of PP2A activity by expression of SV40 small t (ST) maintains BAK phosphorylation after UV damage. Mitochondria were isolated from HCT116-BAK cells expressing vector alone, wild-type ST and mutant ST (C97S) following treatemt ± UV. The level of phospho BAK was determined by solubilising mitochondria in 1% CHAPS buffer and IP was performed using anti p-S117 serum, and immunoprecipitated BAK detected by western blotting with rabbit anti-Bak (Y164, Abcam).

The introduction of specific mutations into BAK, coupled to structural information, has yielded new insights into the interactions between BCL-2 proteins are mediated and identified important sites of contact. For example, previous studies had indicated that a mutation introducing a negatively charged residue into the BH1 helix (residues 125–127 WGR→AEA) resulted in the protrusion of a negative charge into the BAK hydrophobic groove that interfered directly with BH3 protein binding and blocked BAK multimerization [14]. In recent experiments transient binding of BH3 domain containing proteins was also found to be dependent on access to the BAK hydrophobic groove [29]. We hypothesized that phosphorylation of a residue located close to or in the BAK hydrophobic groove may represent a physiological mechanism that regulates BH3 binding, and that removal of this inhibitory phosphate would be required to permit BH3 helix binding and also BAK multimerization. Based on structural information and phospho-site prediction tools, we reasoned that blockade of the hydrophobic groove preventing BH3 binding could potentially be achieved by phosphorylation of residues S91 or S117 (Figure 3A, B). We were able to confirm phosphorylation of S117 on BAK protein isolated from undamaged cells by direct analysis using tandem mass spectrometry [19], but not on S91 as we were unable to detect this tryptic peptide under the conditions used.

To explore the potential functional significance of S91 and S117 we introduced mutations such that either the residue could not be phosphorylated (S→A), or conversely carried a negative charge (S→E) similar to the G126E mutant in other studies [14] that interferes with protein function (Table 1). The BAK mutants were then expressed in HCT116*^bax−/−bak−/−^* cells and each was found to be stable, localized to the mitochondria and able to undergo N-terminal conformational change following UV treatment (Figure S5A, B) indicating that the mutations had a conformation similar to the wild-type protein in undamaged cells. We performed multimerization studies on each mutant to test whether OA was still able to block this step of BAK activation. Treatment of S91A expressing cells with either UV or etoposide showed that this mutant behaved as the wild-type protein in that it was not able to oligomerize following pre-treatment with OA. However in marked contrast, the S117A mutant was refractory to OA inhibition and formed multimers following exposure to either UV or etoposide (Figure 3C). In reciprocal experiments, the S91E mutant formed oligomers following treatment with either UV or etoposide, whereas oligomerization of the S117E mutant was severely impaired after exposure to either agent (Figure 3D). Although equal amounts of BAK were subjected to cross-linking, we noted again that BMH interfered with the detection of monomeric BAK. It is formally possible that slight perturbations in the tertiary structure caused by the introduction of the negative charge may affect the degree of cross-linking. As detailed above, to confirm that the S117E mutant was defective in oligomerization we used identical mitochondrial preparations in direct comparisons in which the BMH cross-linker was substituted with BMOE. We found that BMOE did not interfere with the ability of the BAK antibody to recognize the either monomeric or dimeric forms of BAK in agreement with previous studies, allowing us to confirm that S117E was unable to form dimers following UV or etoposide treatment (Figure 3E). It is possible that the inhibitory phosphate moiety on S117 may serve as a common mechanism to regulate BH3 binding and BAK dimerization since this serine residue, but not the equivalent at position 91, is conserved over a wide range of species (Figure S5C).

**Table 1:** Primer sequences.

Name	Sequence
S91A-F	CGACGCTATGACGCTGAGTTCCAGACC
S91A-R	GGTCTGGAACTCAGCGTCATAGCGTCG
S91E-F	CGACGCTATGACGAGGAGTTCCAGACC
S91E-R	GGTCTGGAACTCCTCGTCATAGCGTCG
S117A-F	CAAGATTGCCACCGCTCTGTTTGAGAGT
S117A-R	ACTCTCAAACAGAGCGGTGGCAATCTTG
S117E-F	CAAGATTGCCACCGAGCTGTTTGAGAGTG
S117E-R	CACTCTCAAACAGCTCGGTGGCAATCTTG
E24-25A/F	CTGCCCTCTGCTTCTGCTGCTCAGGTAGCCCAGGAC
E24-25A/R	GTCCTGGGCTACCTGAGCAGCAGAAGCAGAGGGCAG
SV40ST-F	GAGCTAGCATGGATAAAGTTTTA
SV40ST-R	AAGCTAGCTTAGAGCTTTAAATC

Primers for mutagenesis reactions are listed.

To further confirm that BAK underwent PP2A-mediated dephosphorylation at S117 following DNA damage we raised and affinity purified a specific antiserum against pS117 (Figure S6) and used this to IP BAK from mitochondrial extracts ± damage. Mitochondrial fractions from cells, isolated under conditions of phosphatase inhibition, were incubated with purified PP2A ± OA, and the remaining phospho-BAK was then immunoprecipitated using the pS117 serum. The anti-pS117 serum efficiently precipitated BAK, however incubation of the mitochondria with purified active PP2A resulted in less BAK being pulled down, but inclusion of OA prior to PP2A treatment restored the ability of the pS117 serum to pull down BAK (Figure 3F). We next tested whether depletion of PP2A activity directly affected the phosphorylation status of BAK at S117 following DNA damage. In pilot experiments using siRNA to silence expression of either the PP2A A or C subunits, we noted a rapid overall loss of viability that we infer was due to PP2A being an essential enzyme. We therefore examined BAK S117 phosphorylation in cells expressing SV40 ST or the PP2A binding defective mutant (C97S) as described above, since expression ST had no detectable effect on cell viability. Expression of wild-type or mutant ST had no effect on the ability of the anti-pS117 serum to IP BAK from undamaged cells (Figure 3G). However, while BAK was not able to be immunoprecipitated from cells expressing the ST mutant or empty vector following DNA damage, consistent with dephosphorylation of this site observed in previous experiments. In contrast, the anti-pS117 serum was able to efficiently IP BAK from cells expressing wild-type ST irrespective of whether the cells had been damaged or not, indicating that the UV-induced dephosphorylation at this site has been prevented (Figure 3G). Together these results suggest that S117 is phosphorylated in undamaged cells and that removal of this inhibitory phosphate is mediated by PP2A and that this is required for BAK activation to proceed leading to multimer formation.

### PP2A dephosphorylation allows access of BH3 peptides to the BAK hydrophobic groove

**Figure 4:**
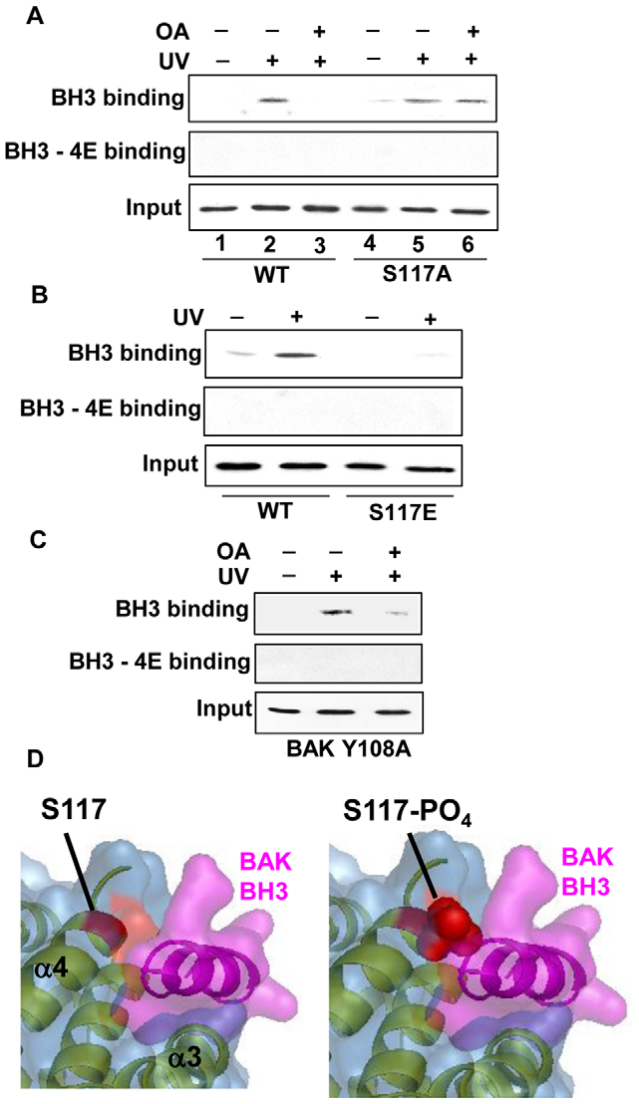
Pull down of BAK by biotinylated BH3 peptides. (**A**) Pull-down experiments were performed with biotinylated BAK BH3 peptide, 20 μM, (top panel) using lysates prepared from HCT116-BAK cells (lanes 1−3) or lysates from S117A mutant cells (lanes 4−6). Pull-downs were also performed with mutant BH3 peptide (BH3-4E) using the same extracts from HCT116-BAK and -S117A (middle panel). Bottom panel shows 5% input of the cell extracts used for pull-down assays. Lysates prepared from both HCT116-BAK and -S117A cells were untreated (lanes 1 and 4), treated with UV (lanes 2 and 5) and treated with OA for 30 min before UV (lanes 3 and 6). BAK was detected by immunoblotting using the Y164 monoclonal. In all panels, 5% input from the cell extract was and blotted for BAK to ensure equal loading. (**B**) Similar pull-down was performed using cell lysates from wild-type BAK or the S117E mutant cells with either BH3 or BH3-4E mutant peptides. The BAK BH3 peptide was unable to pull down the S117E protein. BAK was detected by immunoblotting using the Y164 monoclonal as above. (**C**) BH3 binding experiments were performed using extracts from cells expressing the BAK-Y108A mutant that had been treated ± UV and ± OA. This showed that the Y108A mutant behaved in a similar manner to wild-type BAK, indicating that tyrosine dephosphorylation at Y108 occurred prior to BH3 binding. BAK was detected by immunoblotting as above. (**D**) PyMol model structure of a BAK monomer with a BAK BH3 helix (left) and interference of binding by phosphorylated S117 (right). Model based on PDB structures of BAK (2IMS) and Bim-BH3 peptide bound to BCL-XL (3FDL), Bcl-2 (2VM6) and MCL-1 (2KBW), where the backbone of the pro-apoptotic protein has been replaced by that of BAK.

Since BAK activation involves the binding of BH3-only proteins to the hydrophobic groove and dimerization BAK-BH3:BAK-groove interactions, we reasoned that either the phosphorylation or a negative charge at residue S117 was likely to be a key determinant of BH3 binding. Addition of peptides containing BH3 sequences to purified isolated mitochondria has been shown previously to induce release of cytochrome *c*, [7], [30] and that this is dependent on the accessibility of the hydrophobic BAK binding surface [14]. We modified this *in vitro* assay system to investigate whether either the inhibition of dephosphorylation of S117, or the charge status of the residue, was a key determinant in allowing the binding of BH3-only peptides to BAK. A biotinylated BAK BH3 peptide was used in pull-down assays (Table 2), where it was able to pull down full-length wild type BAK from DNA damaged cells, but that the interaction was prevented by pre-treatment of the cells with OA (Figure 4A). As expected, a BAK peptide containing mutations previously shown to be important for mediating interactions with the hydrophobic surface, (BH3-4E), failed to associate with BAK in these assays. In contrast, the BAK BH3 peptide was able to pull down the BAK S117A mutant following DNA damage even after OA treatment (Figure 4A), whereas the S117E mutant was never able to associate with the BAK peptide (Figure 4B). As the initial step of BAK activation involved tyrosine dephosphorylation at Y108, we reasoned then that mutation of this residue would not impinge upon BH3 binding. Consistent with this idea, the biotinylated BAK BH3 peptide was able to pull down BAK from Y108A expressing cells after DNA damage and this was inhibited by OA treatment (Figure 4C), supporting our data that BH3 binding occurs later in the BAK activation process than Y108 dephosphorylation. Modelling the interaction of a BAK BH3 peptide with a BAK monomer suggested that a phosphate group on S117 would interfere directly with the ability of the BH3 peptide to associate with the hydrophobic BAK groove (Figure 4D). The data presented here demonstrate that the ability of BH3 helices to interact with the BAK hydrophobic groove to initiate dimer and multimer formation during apoptosis is both actively regulated and is likely to be physiologically important.

**Table 2:** Peptides.

Name	Sequence
Bid BH3	IIRNIARHLAQVGDSMDRSIPPGGLV
Bid BH3-4E	IIRNEARHEAQEGDSEDRSIPPGGLV
BAK BH3	TMGQVGRQLAIIGDDINRRYDSEFQT
BAK BH3-4E	TMGQEGRQEAIEGDDENRRYDSEFQT
BAK S117-P	C-TKIAT-pS-LFESG

Sequences of BH3 containing peptides used for pull down experiments are shown. Each peptide was biotinylated at the N-terminus.

For generation of antibodies for phosphorylated serine 117 (S117), phospho- and non-phospho derivatives were synthesised of the BAK sequence surrounding S117. An N-terminal cysteine was added to enable coupling to resin for column purification of phosphor-specific antibodies.

### Dephosphorylation of BAK at serine 117 is required for cytochrome *c* release and apoptosis induction

**Figure 5:**
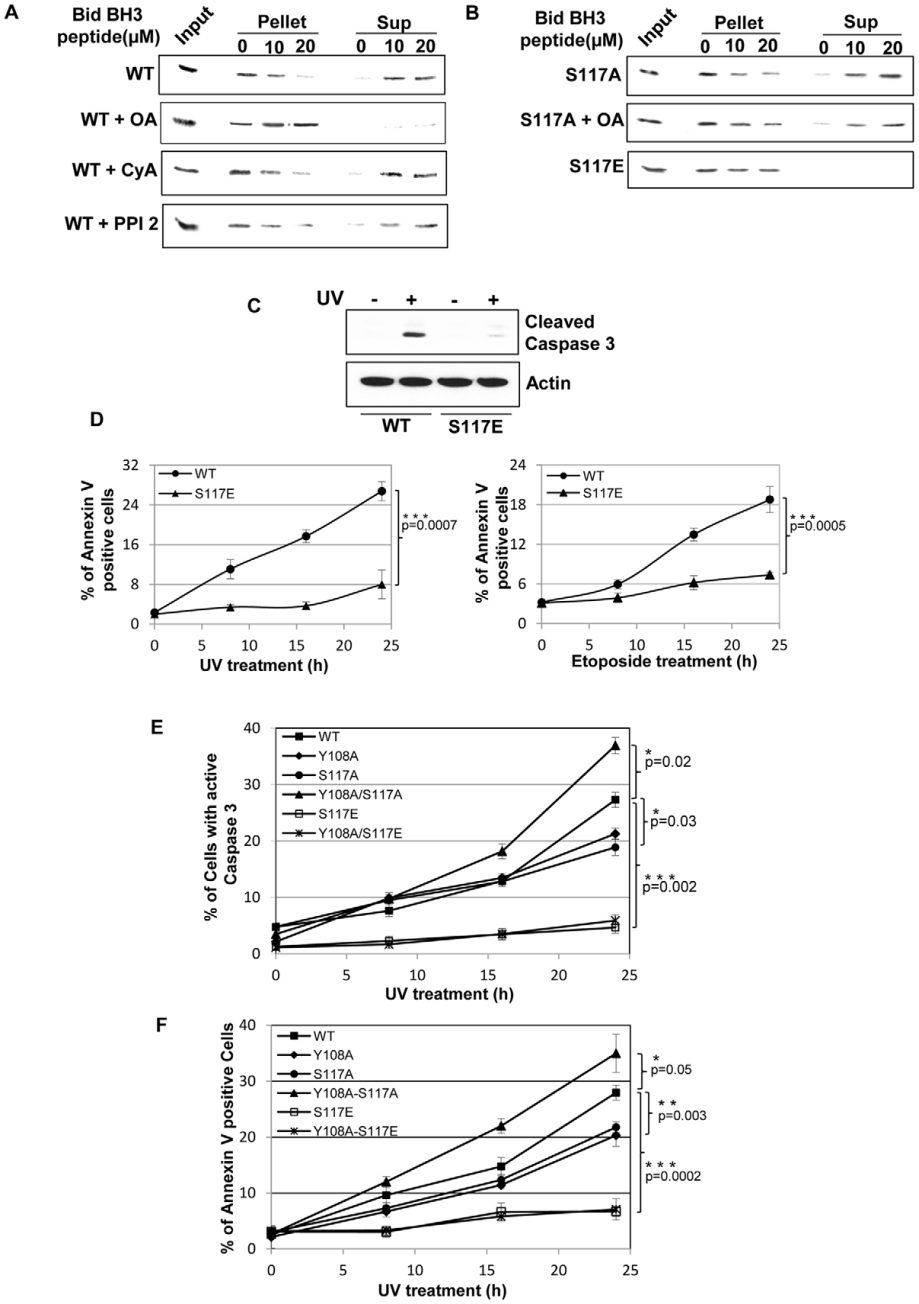
Cytochrome *c* release by a Bid BH3 peptide on mitochondrial preparations treated with phosphatase inhibitors. (**A**) Mitochondrial extracts isolated from HCT116-BAK cells were incubated with Bid BH3 peptide (10–20 μM) and separated into pellet and supernatant fractions by centrifugation. Cytochrome *c* levels were analysed by immunoblotting in both pellet (retention in mitochondria) and supernatant (Sup, released from mitochondrial) fractions. Extracts from cells prepared with prior treatment with OA, but not CyA or PPI-2, were resistant to Bid-mediated cytochrome c release. Mitochondrial and cytosolic fractions were free from cross contamination as assessed by immuoblot (Figure S7). In all cytochrome *c* release assays, an equal amount of isolated mitochondria not treated with peptide was used as input. (**B**) Similar cytochrome *c* release experiments to those in (A) were performed with mitochondrial fractions from S117A mutant cells (top) and S117A mutant cells that had been treated with OA for 30 min (middle). OA was unable to prevent cytochrome *c* release from S117A cells, while S117E mutant cells failed to show cytochrome *c* release when challenged with Bid peptide (bottom). (**C**) Caspase3 activation was determined by immunoblotting in lysates prepared from cells expressing wild-type BAK and the S117E mutant cells treated ± UV. UV treatment resulted in activation of caspase 3 in cells expressing wild-type (WT) BAK, while no activation was seen in S117E cells. (**D**) Cell death was quantified by FACS using annexin-V staining at the indicated times after exposure to UV and etoposide. Treatment with either UV or etoposide resulted in an increase in cell death in HCT116-BAK wild-type (WT) cells, in contrast S117E cells were resistant to cell killing by these agents. Data are the mean percentage of annexin-V positive cells from 3 independent experiments ± S.E.M. (**E**) Activation of caspase 3 in HCT116-BAK wild type (WT) or cells expressing the indicated BAK mutant was determined by FACS using an antibody that specifically recognises only cleaved (therefore active) caspase 3. Data are the mean percentage of caspase 3 positive cells from 3 independent experiments ± S.E.M. (**F**) Cell death in HCT116-BAK wild type (WT) or cells expressing the indicated BAK mutant was quantified by FACS using annexin-V staining at the indicated times after exposure to UV. Data are the mean percentage of annexin-V positive cells from 3 independent experiments ± S.E.M.

Having noted that S117 status was critical for the binding of BH3 sequences to BAK, we then tested whether the release of cytochrome *c* from isolated mitochondria and induction of apoptosis was dependent on the phosphorylation or charge status of BAK at S117. A Bid BH3 peptide was able to cause release of cytochrome *c* from mitochondria that were isolated in buffers lacking phosphatase inhibitors, in line with previous studies (Figure 5A), whereas a peptide having mutations in four residues critical for association failed to release cytochrome *c* from isolated mitochondria (Figure S8). This we infer was likely to be due to the promiscuous activity of phosphatases released from cellular compartments during cell lysis (AA, JF and AS, personal observations). Pre-treatment of cells with OA, but not CyA or PPI-2, blocked cytochrome *c* release in response to challenge with the Bid BH3 peptide (Figure 5A). In agreement with our previous findings, OA was unable to block the release of cytochrome *c* from mitochondria isolated from S117A reconstituted cells by the Bid BH3 peptide, either with or without OA pre-treatment (Figure 5B). In the reciprocal experiment, the BH3 peptide was unable to release cytochrome c from mitochondria of S117E cells (Figure 5B), a finding that is entirely consistent with a previous report where the mutation G126E in the BH1 domain of BAK blocked multimer formation in response to tBid [14].

To test the functional significance of these observations, we analyzed caspase 3 activation in either wild-type or S117E BAK HCT116*^bax−/−bak−/−^* cells and found that activation of caspase 3 was greatly reduced in S117E cells when compared to cells expressing wild-type BAK (Figure 5C). Further, we were able to show that following DNA damage by either UV or etoposide, cell killing as determined by Annexin V positivity, was dramatically reduced in cells expressing the BAK S117E protein when compared to wild type (Figure 5D). These results further support a role for dephosphorylation of S117 as being important for caspase 3 activation and subsequent cell killing.

We supposed then that removal of the inhibitory phosphorylations at Y108 and S117 would lead to enhanced caspase 3 activation and cell killing. While the S117A BAK mutant displayed similar levels of caspase 3 activation and Annexin V positivity compared to cells expressing wild-type BAK, enhanced caspase 3 activation and Annexin V was noted in the Y108A/S117A cells (Figure 5E,F). The S117E mutation, either alone or in combination with Y108A, resulted in a dramatic decrease caspase 3 activation and Annexin V positivity when compared to wild-type cells (Figure 5E,F). These results are in line with previous findings that a temporal series of dephosphorylations are required to complete the BAK activation process and induce cell death.

### BAK conformational change induced by p53 does not depend on S117 dephosphorylation

The p53 protein has been reported to be able to bind to BAK at a site distinct from the hydrophobic groove, involving residues Glu24, Glu25 and Arg160, causing it to undergo N-terminal conformational change [11]. Since the HCT116 cells used here contain wild-type p53, we reasoned that the failure to inhibit BAK conformational change by PP2A inhibition through OA treatment, or in the BAK S117 mutant, was due to p53 activation.

**Figure 6:**
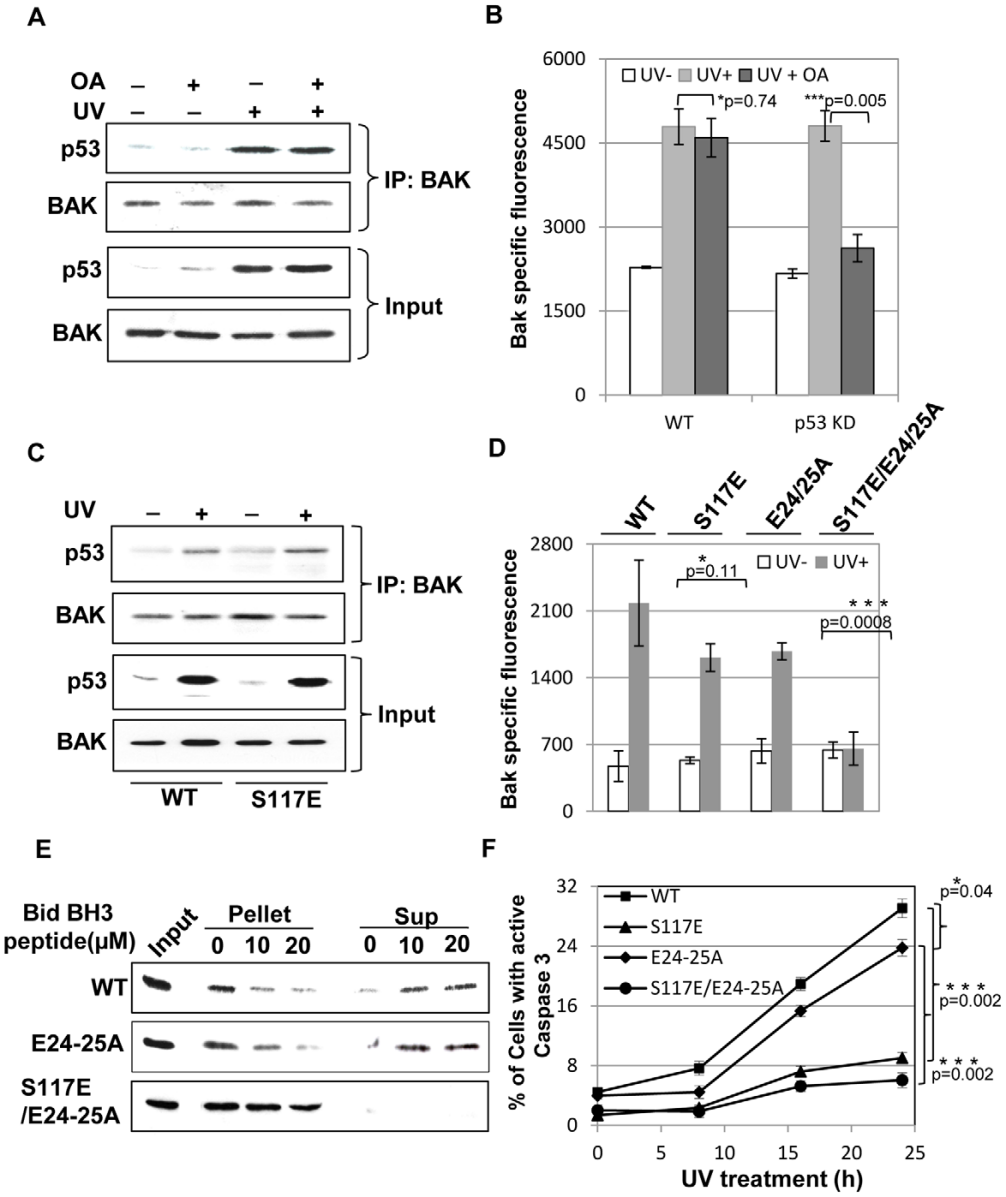
p53- or BH3-mediated BAK conformational change occurs by independent routes. (**A**) BAK was immunopecipitated from HCT116-BAK cells following DNA damage by UV, precipitated protein complexes were then immunoblotted for p53 using DO-1 antibody. Following UV treatment, p53 was co-precipitated with BAK and the complex was not disrupted by incubation of the cells with OA. Input levels of p53 and BAK used in the IP experiments are shown. (**B**) HCT116-BAK cells underwent N-terminal conformational change resulting in an increase in BAK Ab1 specific fluorescence following UV damage that was not inhibited by treatment with OA. However, HCT116-BAK cells in which p53 expression was silenced using siRNA (p53 KD), OA treatment blocked BAK activation in response to UV (n = 3, error bars ±S.E.M.). (**C**) Immunoprecipitation of p53 from S117E cells showed that the mutant interacted with p53 to a similar extent to the wild-type (WT) BAK protein protein. (**D**) N-terminal BAK conformational change as determined by FACS analysis to detect an increase in BAK-specific Ab-1 fluoresence upon UV treatment. BAK activation was reduced but not abolished by mutations S117E or E24-25A when compared to wild-type BAK. In contrast, mutation of the BAK p53 binding site in combination with the BH3 binding site in the S117A/E24-25A mutant could not undergo N-terminal conformational change, suggesting that two distinct regions of BAK can effect conformational change by separate routes. n = 3, error bars ± S.E.M. (**E**) Mitochondrial enriched fractions were prepared from HCT116-BAK cells or cells expressing the indicated BAK mutants in the absence of phosphatase inhibitors in buffers. Preparations were then incubated with the indicated amount of Bid BH3 peptide as before. Cytochrome *c* retained in mitochondria (pellet) or released (Sup) was detected by immunoblotting. (**F**) Caspase3 activation was determined by FACS analysis of cells expressing wild-type BAK and the indicated BAK mutant treated ± UV. UV treatment resulted in activation of caspase 3 in cells expressing wild-type (WT) BAK or the E24-25A mutant, while no activation was seen in S117E or S117E/E24-25A cells (n = 3, error bars ±S.E.M.).

To test this idea, we first confirmed that p53 was able to interact with BAK following UV damage ± OA treatment. In Co-IP experiments where comparable amounts of BAK were immunoprecipitated, we find that OA treatment had no effect on the BAK-p53 association (Figure 6A). Knock-down p53 expression by siRNA silencing confirmed that this prevented an increase in p53 levels caused by UV damage (Figure S9A). In the p53-silenced cells UV was still able to cause BAK N-terminal conformational change as judged by increased Ab-1 BAK-specific fluorescence following UV damage (Figure 6B). In contrast, OA treatment of p53-silenced cells inhibited BAK conformational change following DNA damage (Figure 6B, S10A). Further, the BAK S117E mutant, that we showed undergoes N-terminal conformational change, associated with p53 following DNA damage to levels equivalent to the wild-type protein (Figure 6C), supporting the idea that p53 can activate BAK by a mechanism separate from that used by BH3 proteins interacting with the hydrophobic groove. This implies that BAK conformational change would be inhibited by a combination of mutations that abolish simultaneously both p53 and BH3 protein binding. Indeed, while the individual BH3-binding or p53-binding defective mutants were each able to undergo N-terminal conformational change, the S117E/E24-25A triple mutant that can no longer interact with either BH3 or p53 proteins failed to undergo conformational change after UV treatment (Figure 6D, S10B). We attribute the slightly lower magnitude of the response to small differences in expression levels of the mutants. These results demonstrated that p53 was able to produce a change in the conformation of BAK even when access to the BH3 groove is impaired, indicating that two distinct routes can be employed to alter BAK conformation. Consistent with this, we found that the E24-25A mutant was still able to release cytochrome *c* from mitochondria challenged with a Bid BH3 peptide, and that cytochrome *c* release was inhibited by further mutation of S117 in the S117E/E24-25A triple mutant (Figure 6E). Access to the BH3 binding groove to promote cytochrome *c* release was directly related to their ability to activate caspase 3, where we found that either the S117E or S117/E24-25A mutant were defective in the ability to activate caspase 3 while the E24-25A mutant retained this function (Figure 6F). Altogether, these findings demonstrate that access to the BAK hydrophobic groove is critical to mediate cell killing, but conformational change can be brought about via two distinct mechanisms.

## Discussion

The present work has uncovered an important new step in our understanding of how signalling pathways are intimately linked to conformational changes in BAK that result in MOMP and cell death. Activation of BAX and BAK during the induction of apoptosis has been intensively studied, with details of the mechanisms underlying these processes emerging over the past few years that suggest some similarities, yet also clear fundamental differences between how the two proteins respond to apoptotic signals. It is apparent however that both proteins act at a nodal point during apoptotic commitment, in that their oligomerization generates pores in the mitochondrial membrane releasing apoptotic factors representing a ‘point-of-no-return’ in the apoptotic cascade. Together with our previous results our new data provide important insights into a novel and potentially physiologically significant post-translational mechanism regulating BAK activation.

This work strengthens the idea that conformational changes in BAK leading to its multimerization are intimately linked to, and indeed dependent upon a temporal series of dephosphorylations. Recently we showed that a specific tyrosine dephosphorylation at residue 108 constituted the initial step in BAK activation that was required for all subsequent steps in BAK activation leading to cell killing. We find that a second dephosphorylation mediated by PP2A is critical to allow binding of BH3 helices and permit BAK:BAK interactions for multimerization, cytochrome *c* release from mitochondria, caspase activation and apoptotic cell death. At present we have only had the opportunity to examine S117 phosphorylation in our HCT116-BAK system. Our previous 2D gel-western blot studies indicated BAK was heavily phosphorylated in undamaged cells with ∼10 residues likely to be phosphorylated, but as only 1–2 spots were detected this indicates a population of BAK molecules that were fairly homogeneous. Further investigations to determine the stoichiometry of S117 phosphorylation in other cell types may be warranted to determine how widespread this mechanism might be, and also whether the equilibrium between phosphorylated/non-phosphorylated BAK at S117 can be affected by differing PP2A expression levels and profiles of subunit expression that are likely to occur between cell types.

**Figure 7:**
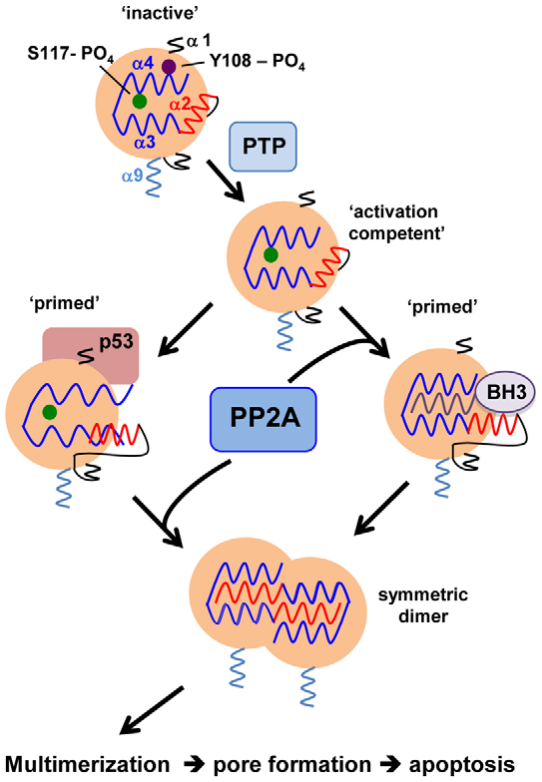
Proposed model of BAK multimerization integrating dephosphorylation with conformational changes triggered by BH3 or p53 proteins. Based on our findings we propose a model of BAK activation in which two inhibitory phosphate moieties must be removed to permit conformational change that can be triggered by either BH3-only or p53 proteins, which is then followed by multimerization. Apoptotic stimuli activate protein tyrosine phosphatases (PTP) that remove the phosphate group from Y108 to generate what we term the ‘activation competent’ form of BAK, which is then able to undergo conformational change to generate the ‘primed’ form. The primed form of BAK can, we propose, be generated by two independent routes that involve either the binding of BH3-only proteins to the hydrophobic groove, or binding of p53 to a site on the α1 helix. Generation of the primed BAK form by BH3 proteins first requires PP2A-dephosphorylation of S117 to permit transient binding of BH3 proteins and also subsequent insertion of a BAK BH3 helix for dimer formation. Alternatively, BAK dephosphorylated at Y108 can recruit p53 directly to undergo conformational change, but still requires PP2A-dephosphorylation at S117 to nucleate dimer formation.

Both BAX and BAK are known to undergo a series of conformational changes that can be induced by the binding of BH3 helices to hydrophobic surfaces on each molecule. In contrast to BAX, BAK does not possess a binding site for BH3 proteins at the N-terminus and the α9 helix is inserted into the mitochondrial membrane. Thus the α9 helix does not block access to the hydrophobic BH3-binding surface involving the BH1 domain, thereby potentially leaving BAK susceptible to aberrant activation by BH3 proteins. In this scenario inappropriate activation of BAK would have detrimental consequences for cell survival, so it is likely that other mechanisms restrict BAK activation to ensure that activation only proceeds following multiple positive signals generated by an apoptotic stimulus. Based on our current findings we propose a model of BAK activation in which dephosphorylation at S117 is coupled to BH3 binding and multimerization (Figure 7). In our model the phosphate group on S117 effectively blockades the BAK hydrophobic groove to prevent the binding of a BH3 helix. Thus, S117 phosphorylation can act in two ways. First, it can prevent triggering of BAK conformational change by binding of BH3 helices from BH3-only proteins, and second, it prevents insertion of the BAK BH3 helix into another BAK molecule that nucleates dimerization. Phosphorylation at S117, we conclude, hinders access of a BH3 helix to the BAK binding surface, a mechanism that is believed to be a primary mode of interaction amongst different BCL-2 family proteins. An important corollary to our findings then is that BAK dephosphorylation at S117 may be a pre-requisite for the inhibitory effect of pro-survival proteins, mediated by binding MCL-1 or BCL-X_L_ for example, to be exerted, or alternatively the triggering of BAK conformational change by BH3-only proteins. Thus, the sustained phosphorylation we observe in healthy cells indicates a further level of regulation that restrains BAK activation in addition to the binding and inhibitory effects of MCL-1 or BCL-X_L_. Whether the S117 phosphorylation status impinges upon MCL-1, BCL-X_L_ or BH3-only protein binding to a similar degree merits further detailed investigation to unravel the complexities raised by our findings.

A number of studies report that binding of p53 can trigger BAK activation, and BAK residues required for p53 interaction have been identified to the N-terminal region of the protein [9], [11], [31], [32]. Consistent with our model, we find that indeed p53 can trigger BAK conformational change and this can occur independent of the phosphorylation or charge status of S117. However, as demonstrated by the mutational and inhibitor studies, conformational change triggered by p53 is still dependent on removal of the negative charge at S117 that permits BAK dimerization for any effects on cell death.

PP2A has been demonstrated in many studies to have tumour suppressor activity. Regulatory B subunits of PP2A are believed to be involved in targeting of PP2A to selected substrates. Using specific shRNA constructs, we provide evidence implicating the involvement of the PP2A B56αsubunit in BAK activation and subsequent cytochrome *c* release following DNA damage. BAK can now be considered as an important PP2A target, revealing a novel direct link between PP2A and the apoptotic machinery. These findings may help to explain in part why reduced levels of PP2A activity, that have been found in human cancers [28], [33], may contribute towards tumour initiation or maintenance by helping to confer apoptotic resistance through impaired BAK activation. Noteworthy also are reports in model cell culture systems indicating that inhibition of PP2A activity was linked to cell transformation [25].

In summary, we have shown that BAK multimerization requires removal of inhibitory phosphorylation at the hydrophobic groove, mediated by PP2A, in the stepwise process of activation. Both BH1 and BH3 domains have been shown to be important for homo-oligomerization of BAK that involves an interaction between the BH3 domain of one monomer and the canonical binding pocket on another BAK molecule. Our results indicate that BAK activation involving the binding of BH3 or p53 proteins to BAK activation is intimately linked to phosphatase signaling pathways that regulate protein-protein interactions. The identification of a second dephosphorylation event on BAK that is also critical to enable BAK activation to proceed to completion, reveals a temporal series of dephosphorylation events governs the activation potential of BAK. While BAK post-translational modifications add complexity to the emerging picture of the process of BAK activation, they not only pose new challenges in our understanding of these critical events but also provide wider opportunities to directly regulate BAK activity to modulate cell death in a number of important human diseases involving aberrant apoptotic processes.

## Materials and Methods

### Plasmid Constructs

BAK mutants (S91A, S91E, S117A, S117E, E24/25A, S117E/E24/25A) were generated by PCR mutagenesis using QuickChange XL site-directed mutagenesis kit (Stratagene) using pLSXN-BAK as a template. Mutations were confirmed by DNA sequencing. Primer sequences for mutations are listed in Table 1. Both WT SV40 ST and mutant SV40 ST (C97S) were cloned into pF1005 [34] by PCR amplified from pcDNA3-WT SV40 ST and pcDNA3-mt SV40 ST (C97S) constructs respectively. Both the constructs were verified by DNA sequencing. shRNA-GFP vectors for PP2A B56α and B56γ subunits, together with empty vector pMKO.1 DNA were supplied by Addgene, plasmids 10676, 10680 and 10681 [28].

### Cell Culture and retroviral infection

HCT116*^bax−/−bak−/−^* double knockout (DKO) cells were kindly provided by R. Youle [35]. Cells were cultured in DMEM supplemented with 10% FCS and glutamine. Cells were transfected with plasmid DNA using Fugene 6 (Roche, Germany) and selection was carried out using G418 (Sigma). Retroviruses were produced as described previously in PT67 cell lines [19]. BAK constructs were reconstituted in human colorectal cancer cell lines HCT116*^bax−/−^* ^bak−/−^ cells by retroviral infection and selected by G418. HCT116-BAK cells were co-transfected with both pF1005-WT ST and pF10005-mt ST(C97S) and selection was carried out using puromycin. shRNA plasmids for PP2A B56α and γ subunits were transfected into double knockout cells using polyethylenimine (Polyscience). Mitotracker deep red FM was from Molecular Probes (Invitrogen).

### Chemicals

Okadaic acid, Cyclosporin A and Sodium Stibogluconate were purchased from Calbiochem, Fostriecin, PPI-2, Etoposide and Camptothecin were from Sigma.

### FACS analysis of BAK conformation

BAK N-terminal exposure was detected by flow cytometry using conformation-specific antibody Ab1 (Calbiochem) ± exposure to DNA damage in presence of inhibitors according to published procedures [19], [36]. Briefly, following drug treatment or UV damage cells were fixed in paraformaldehyde (0.25% PFA/PBS) for 10 minutes at room temperature. Cells were harvested by scrapping, washed with PBS. Cells were permeabilised with 0.01% saponin/PBS and incubated with BAK Ab-1 primary antibody (AM03; Calbiochem), BAK Ab-2 primary antibody (AM04; Calbiochem) or mouse IgG1 (Pharmingen) for 30 minutes at 4°C. Cells were then washed and incubated with rabbit anti-mouse phycoerythrin or with goat anti-mouse Allophycocyanin secondary antibody for 30 minutes at 4°C. Cells were washed and resuspended in PBS for analysis with a CyAn ADP Analyser (Beckman). 10,000 cells were analysed per sample. To quantitate the flow cytometric results using Ab-1 the data was then manipulated as previously described [19], [36]. Briefly, cells exhibiting a light scatter profile associated with apoptotic cells were gated out and the median BAK-associated fluorescence was determined by subtracting the median fluorescence of the parallel IgG control from each test sample. The median value was then multiplied by the percentage of BAK positive cells as determined by the IgG control to give the Ab-1 BAK-specific fluorescence of each sample. Cell death was quantified by flow cytometric analyses using Annexin-V AlexaFlour 647 conjugate (Cambridge Bioscience), and active caspase 3 using FITC-conjugated antibody (BD Pharmingen).

### Immunoprecipitation

For immunoprecipitation studies of phospho-BAK total cell lysates were prepared in IP buffer (20 mM Tris-HCl pH 7.4, 135 mM NaCl, 1.5 mM MgCl_2_, 1 mM EGTA, 10% glycerol, protease inhibitor cocktail) containing 1% CHAPS and incubated on ice for 30 min. The supernatants containing protein extracts were collected by centrifugation at 12,000 rpm for 10 min. The antibody was then added to the extract and incubated overnight at 4°C with rotation. 30 μl of a 50% slurry of either protein-A shepharose or protein G – shepharose were added to the samples and incubated at 4°C for 90 min. The beads were then washed in IP buffer for 3 times and dissolved in 25 μl of 2X SDS buffer. For interaction between p53 and BAK, immunoprecipitations were performed from mitochondrial fractions solubilized in IP buffer.

### Cytochrome *c* release from isolated mitochondria

Mitochondria were isolated in AT buffer (300 mM Trehalose, 10 mM HEPES-KOH pH 7.7, 10 mM KCl, 1 mM EGTA, 1 mM EDTA and 0.1% BSA) as described [37]. Cytochrome *c* release experiments were performed with 25 μg of either frozen or freshly isolated mitochondria in AT buffer containing 80 mM KCl. The mitochondria were incubated with 5–10 μM of Bid BH3 peptide for 30 min at 37°C. To measure cytochrome *c* release samples were centrifuged at 10,000 rpm for 10 min at 4°C and the pellet and the supernatant fractions were analyzed by immunoblotting.

### Immunostaining

The indicated cells were grown on coverslips for 24 hrs followed by transfection with pMKO.1 empty vector or shRNA constructs for B56α or B56γ. After 48 hrs, cells were treated with etoposide for 24 hrs. For Mitotracker staining, cells were treated with 200 nM of Mitotracker deep red FM (Invitrogen) for 30 min in PBS at 37°C. After 3 washes in PBS, cells were then fixed with 4% paraformaldehyde in PBS for 10 min and washed with PBS. The fixed cells were permeabilized with 0.15% Triton X-100 for 15 min at RT and blocked in 3% BSA for 1 hr. The anti cytochrome *c* antibody (clone 6H2.B2) was used at 1∶100 for 2–3 hr at RT in 1% BSA and followed by incubation with an Alexa-Flour 546 goat anti-mouse secondary antibody (Invitrogen) for 1 h. Cover slips were mounted with Fluoromount-G (Southern Biotech) containing DAPI. Images were obtained by confocal laser scanning microscopy (Zeiss LSM510) using a 488 nm Argon II laser (green fluorescence), a 561 nm DPSS561-10 laser for cytochrome c, a 405 nm Diode and a 633 nm HeNe laser (far red fluorescence).

### Generation of phospho-S117 antisera

The antibodies were generated by BioGenes GmbH, Germany. Briefly, rabbits were immunized three times with synthetic phospho-S117 peptide (Table 2). Serum was affinity purified through S117 specific phospho- and non-phospho columns. Peptides were synthesized and purified by HPLC by K. Di Gleria of the peptide synthesis facility at the WIMM.

### Immunoblot analysis and antibodies

Cell lysates were prepared in either RIPA buffer or IP lysis buffer (1% CHAPS buffer) containing complete mini protease inhibitor tablet (Roche) and phosphatase inhibitor cocktail 1 and 2 (Sigma) as required. For detection of cleaved caspase 3, cells were lysed in PBS containing 1% Triton X-100 supplemented with protease inhibitor cocktail and phosphatase inhibitor cocktail. 20–30 μg of proteins were separated on SDS-PAGE and transferred to Hybond-C membranes (Amersham Biosciences). Standard procedures were applied for western blotting. The following antibodies were used for immunoblotting: rabbit monoclonal anti BAK (Y164, abcam), mouse monoclonal BAK Ab1 (TE-100, Calbiochem), mouse monoclonal BAK Ab2 (Oncogene), anti cytochrome c (BD Pharmingen^TM^, clone 7H8.2C12 and clone 6H2.B2), anti p53 (DO1, CRUK), anti Cox IV (abcam), anti α-Tubulin (Calbiochem), anti SV40 small T antigen (PAB280, Calbiochem), anti PP2A- B56α and B56γ (Santa Cruz), anti actin (abcam).

### PP2A phosphatase assay

Mitochondria were isolated as described above and washed three times in phosphatase buffer (50 mM Tris-HCL, pH 7.5, 1 mM MnCl_2_, 1 mM DTT, 10 mM MgCl_2_, 0.2 mM EDTA, 0.2 mM EGTA, protease inhibitor cocktail). Mitochondria were dissolved in phosphatase buffer and aliquoted into 3 fractions. One fractions was incubated with okadaic acid for 30 min at 37°C prior incubation with purified PP2A (PP1/PP2A Toolbox kit, Millipore) at 30°C for 30 min. All mitochondrial fractions were then washed and solubilised in 1% CHAPS buffer. Immunoprecipitation were performed with IgG, rabbit anti BAK ab (Y164) or anti phospho-S117 serum then western blotted for BAK using Ab1.

### ELISA

Microtitre plates were coated with peptide at 5 μg/ml in borate buffered saline (BBS, 0.1 M boric acid, 25 mM sodium tetraborate, 75 mM NaCl, pH 8.5) for 24 hrs at 4°C, then blocked with BBS containing 1% BSA for 1 hr at room temperature. Antibodies were diluted in BBS/1% BSA and incubated with peptide for 2 hrs at room temperature, then washed 3x in Tris buffered saline (TBS) containing 0.05% Tween 20 (TBS-T). Secondary antibody (goat anti-rabbit HRP conjugate, Dako) was diluted 1/500 in BBS/1% BSA, then added to the wells and incubated for 1 hr at room temperature. The wells were washed 3x in TBS-T, before the addition of substrate (TMB, Sigma). Colour reactions were allowed to develop for 5 mins then stopped by the addition of 25 μl H_2_SO_4_. Absorbance was measured at 450 nm with a reference wavelength at 630 nm.

### Isolation of mitochondria and BAK multimerization assay

Cells were seeded and treated with phosphatase inhibitors for 30 min before apoptotic stimuli either by 10 mJ/cm^2^ UV or 50 μM Etoposide for 8 hrs. Cells were harvested and washed once in cold PBS. To isolate mitochondria cells were homogenized in HIM buffer (20 mM HEPES-KOH, pH 7.5, 10 mM KCl, 1.5 mM MgCl_2_, 250 mM sucrose, 1 mM EGTA, pH 7.4 supplemented with protease inhibitor cocktail) using a protocol described previously [38]. Briefly, homogenates were centrifuged at 1000 rpm for 5 min at 4°C. The supernatant was collected and centrifuged again at 10,000 rpm for 20 min at 4°C to obtain cytosolic and mitochondrial-enriched fractions. The pellet containing mitochondria was then resuspended in HIM buffer. The protein concentration was measured by Bio-Rad protein assay. 100 μg of mitochondria was resuspended in HE buffer (10 mM HEPES pH 7.5, 1 mM EDTA supplemented with protease inhibitor cocktail) and incubated with 10 mM BMH (1, 6-bismaleimidohexane, Pierce), or BMOE (Pierce) as indicated, at 37°C for 1 hr to cross-link BAK. The reactions were centrifuged at 12,0000 rpm for 10 min and pellets were then mixed in 25 μl of 2× protein sample buffer and subjected to NuPAGE 4–12% gradient gel and western blotting against rabbit human anti-BAK antibody (Y164, abcam). Cells were pre-treated with phosphatase inhibitors as indicated. Final concentrations of inhibitors or DNA damaging agents were as follows: okadaic acid (OA), 7 nM, Fostriecin, 0.5 μM, Cyclosporin A (CyA), 2.5 μM, PPI-2, 5 nM, camptothecin, 6 μM.

### BH3 peptide binding assays

Peptides were synthesized and purified by HPLC by K. Di Gleria of the peptide synthesis facility, WIMM. BH3 peptide binding assays were performed as described previously [39]. Briefly, 1–2 mg of extract was prepared in 1% CHAPS buffer (10 mM Hepes, pH 7.5, 150 mM NaCl, 1% CHAPS, supplemented with EDTA-free protease inhibitor cocktail and phosphatase inhibitor cocktail) was incubated with 10 μM biotinylated peptide for 4 hr with agitation at 4°C. 30 μl of avidin-agarose beads were then added and incubate overnight at 4°C. Beads were then pelleted, washed 3 times in CHAPS buffer and resuspended in 25 μl of 2× loading buffer.

### siRNA knockdown of p53

HCT116-BAK cells were transfected with a pool of 200 nM of p53 siRNA oligo (Dharmacon) by using DharmaFECT4 reagent (Dharmacon) according to the manufacturer's instructions. Cells were also transfected with negative control siRNA (Qiagen). After 48 hours, cells were treated with UV, or OA prior UV damage, and subjected to BAK N-terminal conformational change analysis by FACS as described.[19], [36] The efficiency of p53 knockdown was verified by immunoblotting using the anti-p53 monoclonal DO-1.

## Supporting Information

Figure S1 **BAK N-terminal conformational change is insensitive to PP2A inhibitors.** HCT116-BAK cells were analysed for BAK N-terminal conformational change as described in Refs ^19, 35^. Left panel: Representative histograms of FACS analysis to measure BAK activation using BAK-specific fluoresence as determined by binding of the N-terminal conformational change specific Ab1 antibody, without (−, red) or with (+, green) UV damage. Where indicated phosphatase inhibitors (OA, CyA and PPI 2) were added prior to irradiation. Right panel: quantification of BAK Ab1 specific fluorescence (N = 3, ± S.E.M.).
(TIF)

Figure S2 **BAK multimerization assay in response to DNA damage in presence of PP2A inhibitor OA.** HCT116-BAK cells were treated with DNA damaging agents UV or etoposide. Mitochondrial sub-cellular fractions were prepared and exposed to the indicated cross-linking followed by analysis by immunoblot. BMH was routinely used as cross-linker, or alternatively BMOE as indicated. (**A**) Cells were damaged ±UV and treated ±OA then mitochondrial extracts prepared and divided into two equal parts for cross-linking of BAK, one was treated with BMH and the other with BMOE. In both cases UV treatment induced the formation of multimeric BAK complexes. We note however that BMH sometimes lowers the detection of monomeric BAK even though input levels were the same. Cross-linking using BMOE generates only dimeric BAK complexes rather than the multimers generated by BMOE, in agreement with previous reports (see Ref 16) and does not affect the detection of monomeric BAK species. We conclude that OA efficiently inhibits BAK multimer formation following UV damage. OA also inhibits Bak multimerization using BMH either in response to Etoposide (**B**) or camptothecin (Cpt) treatment (**C**). In all panels immunoblots were performed with monoclonal rabbit anti-Bak (abY164, Abcam). 5% input of mitochondria used in the cross-linking reactions was run as a loading control of BAK levels. All the experiments were repeated at least twice. In all multimerization experiments using BMH as cross-linker monomeric BAK (M) as well as an intramolecularly cross-linked BAK species (M_x_) can be detected. Where indicated, multimers corresponded to the mobility of BAK dimers (D), trimers (T) or higher molecular weight species.
(TIF)

Figure S3 (A) HCT116-BAK cells were transfected with plasmids expressing either wild type SV40 ST or the C97S mutant that does not interact with or inhibit PP2A activity, or empty vector. Immunoblot analyses confirmed expression of either wild type or mutant SV40 ST using anti-ST monoclonal antibody (PAB280). α-Tubulin acted as loading control. (B) Silencing of PP2A-B56α and B56γ subunits by shRNA. Immunoblotting of lysates from cells transfected with either empty vector pMKO.1(EV) or pMKO.1 derivatives expressing specific shRNA for PP2A subunits B56α or B56γ. Remaining B56α and B56γ proteins were detected using specific antisera. Silencing of B56α and B56γ did not alter BAK or cytochrome c levels. Actin represents equal loading control. No  =  non-transfected cells, Vector  =  cells transfected with empty vector.
(TIF)

Figure S4 **Confocal microscopy images of cytochrome c staining in cells treated ± etoposide.** HCT116-DKO (double knockout *bax−/−bak−/−*) or HCT116-S117E mutant cells were fixed and stained for cytochrome c and analysed by confocal microscopy 24 hrs post treatment with etoposide. Mitotracker deep red FM was used to label mitochondria and nuclear DNA stained with DAPI. Both HCT116–DKO and S117E mutant failed to release cytochrome c after treated with etoposide.
(TIF)

Figure S5 **BAK mutants undergo N-terminal conformational change following DNA damage.** (A) BAK conformational change was analysed in wild type (WT), S91A and S117A mutant cells ± DNA damage by UV (−, red, +, green) (left panel). Quantification of the BAK Ab1 specific fluorescence in WT, S91A and S117A (right panel). (B) BAK conformational change was also analysed in S91E and S117E mutants that also underwent N-terminal conformational change similar to wild type BAK (−, red, +, green) (left panel). Quantification of BAK-specific fluoresence (right panel). (C) BAK mutation S117D also impairs multimerization in response to UV damage using BMOE as cross-linking agent. (D) S117 is conserved amongst species.
(TIF)

Figure S6 **Characterization of anti-phospho S117 BAK polyclonal antiserum.** (**A**) ELISA was performed with affinity purified polyclonal rabbit serum raised against a phospho S117 peptide. Reactivity was dependent upon the phospho group on the BAK peptide, rabbit IgG was used as a negative antibody control. (**B**) Specificity of phospho S117 BAK serum. Immunoprecipition (IP) of BAK was performed with phospho S117 serum using extracts from cells expressing either wild type BAK or the BAK S117A mutant (top panel), or wild type BAK or S117E mutant (bottom panel). Following IP, BAK was detected by western blotting with mouse anti-BAK Ab1.
(TIF)

Figure S7 **Purity of mitochondrial fractions.** Fractionations were performed from HCT116-BAK expressing cells either treated ± with phosphatase inhibitors (OA, CyA and PPI 2) as indicated. After fractionation, mitochondrial and cytosolic extracts were assayed by western blot for cross-contamination using either Cox IV as a mitochondrial marker or tubulin as a cytosol specific protein. Mitochondria were then used for either multimerization, cytochrome c release or immunoprecipitation assays.
(TIF)

Figure S8 **Mutant Bid BH3 peptide failed to release cytochrome c from HCT116-BAK expressing cells.** Four conserved hydrophobic residues in the Bid BH3 peptide required for binding to BCL-2 family proteins were mutated to negatively charged glutamate (E) residues (BH3-4E). Cytochrome c release assays were performed with isolated mitochondria incubated with increasing concentrations of the mutant BH3 peptide. Following incubation with the peptide, mitochondria were harvested and cytochrome c retained in the mitochondria (pellet) or released into the supernatant (Sup) was analysed by immunoblot.
(TIF)

Figure S9 (A) siRNA silencing of p53 in HCT116-Bak cells was achieved with specific Oligos (Dharmacon). p53 protein levels were not recovered following UV damage in p53-silenced cells. BAK levels were unaffected by silencing of p53. Cells were also mock treated (m) with transfection reagent only, or transfected with control non-targeting siRNA (c). (B) Immunoblot of the BAK mutants S117E, the p53 binding site mutant E24-25A, or the triple mutant S117A/E24-25A were expressed to similar levels to the wild type protein in HCT116 cells.
(TIF)

Figure S10 (A) FACS analyses of BAK N-terminal conformational change in HCT116-BAK wild type (WT), S117E, E24-25A or S117E/E24-25A cells following treatment ± with UV. The shift in fluoresence by binding of the BAK Ab-1 antibody following UV treatment is shown in green, compared to non-treated cells (red). (B) FACS analyses profile of HCT116-BAK wild type (WT) and cells in which p53 expression was knocked down by siRNA (p53KD) following exposure ± to UV and ± OA treatment. Red indicates no UV treatment, and green the profile from cells treated with UV. All experiments were performed at least 3 times, representative histograms are shown.
(TIF)
