# Supplementary figures and images for: Blockade of the BAK Hydrophobic Groove by Inhibitory Phosphorylation Regulates Commitment to Apoptosis
Source: PLoS One. 2012 Nov 26;7(11):e49601. doi: 10.1371/journal.pone.0049601 (PMC3506661; doi:10.1371/journal.pone.0049601)

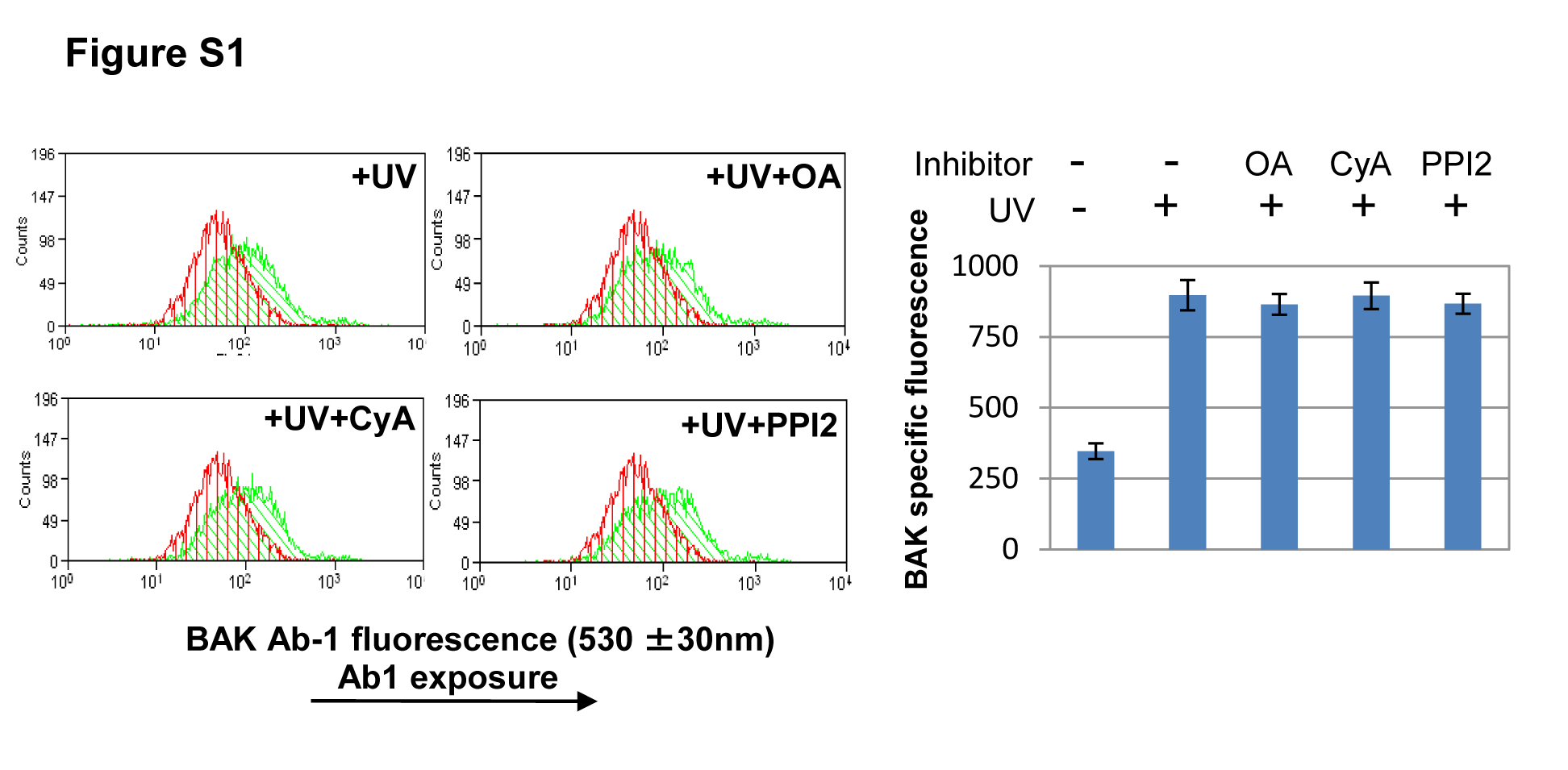

Supplement: Figure S1 — BAK N-terminal conformational change is insensitive to PP2A inhibitors. HCT116-BAK cells were analysed for BAK N-terminal conformational change as described in Refs 19, 35. Left panel: Representative histograms of FACS analysis to measure BAK activation using BAK-specific fluoresence as determined by binding of the N-terminal conformational change specific Ab1 antibody, without (−, red) or with (+, green) UV damage. Where indicated phosphatase inhibitors (OA, CyA and PPI 2) were added prior to irradiation. Right panel: quantification of BAK Ab1 specific fluorescence (N = 3, ± S.E.M.). (TIF) [file pone.0049601.s001.tif]

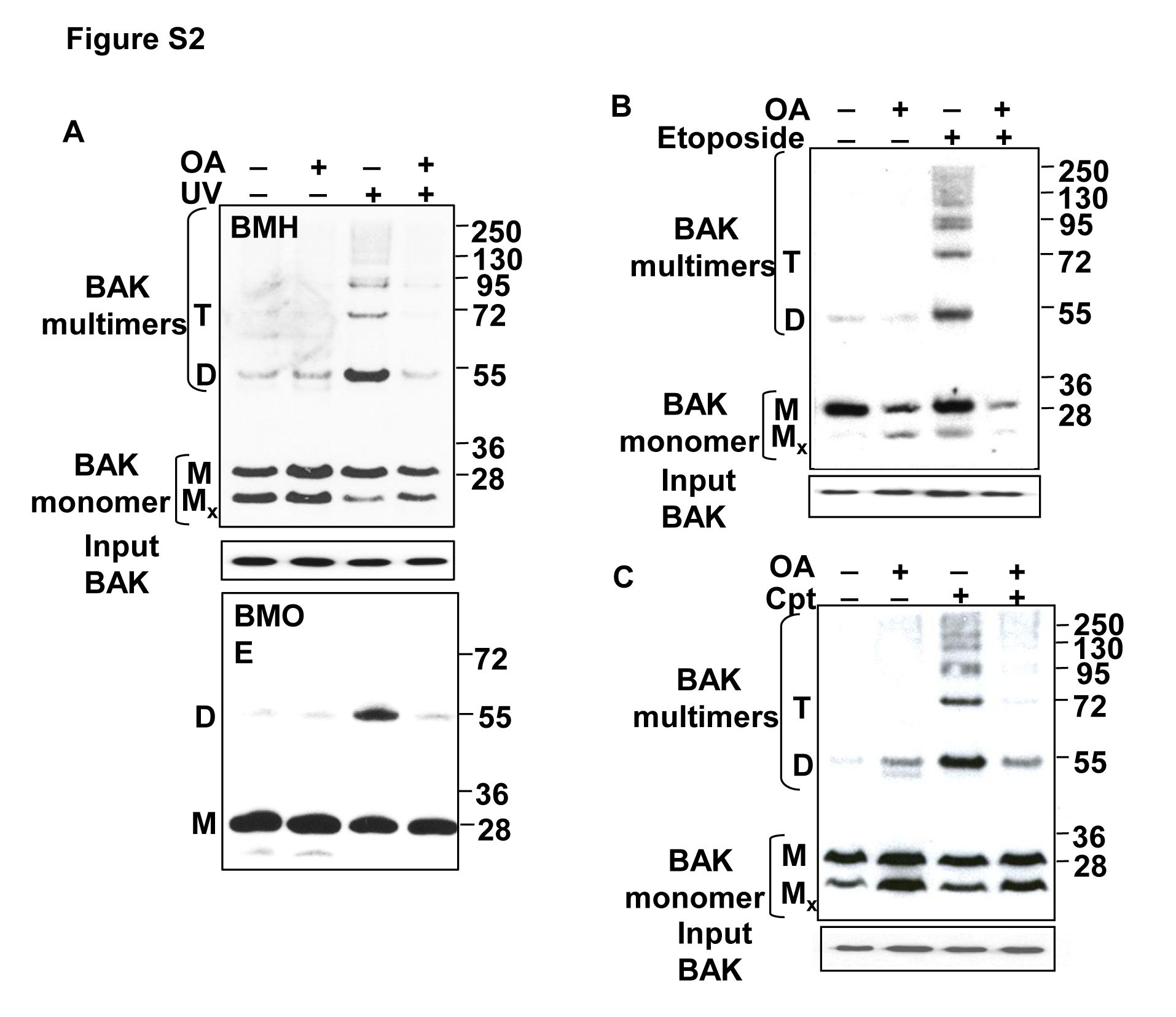

Supplement: Figure S2 — BAK multimerization assay in response to DNA damage in presence of PP2A inhibitor OA. HCT116-BAK cells were treated with DNA damaging agents UV or etoposide. Mitochondrial sub-cellular fractions were prepared and exposed to the indicated cross-linking followed by analysis by immunoblot. BMH was routinely used as cross-linker, or alternatively BMOE as indicated. (A) Cells were damaged ±UV and treated ±OA then mitochondrial extracts prepared and divided into two equal parts for cross-linking of BAK, one was treated with BMH and the other with BMOE. In both cases UV treatment induced the formation of multimeric BAK complexes. We note however that BMH sometimes lowers the detection of monomeric BAK even though input levels were the same. Cross-linking using BMOE generates only dimeric BAK complexes rather than the multimers generated by BMOE, in agreement with previous reports (see Ref 16) and does not affect the detection of monomeric BAK species. We conclude that OA efficiently inhibits BAK multimer formation following UV damage. OA also inhibits Bak multimerization using BMH either in response to Etoposide (B) or camptothecin (Cpt) treatment (C). In all panels immunoblots were performed with monoclonal rabbit anti-Bak (abY164, Abcam). 5% input of mitochondria used in the cross-linking reactions was run as a loading control of BAK levels. All the experiments were repeated at least twice. In all multimerization experiments using BMH as cross-linker monomeric BAK (M) as well as an intramolecularly cross-linked BAK species (Mx) can be detected. Where indicated, multimers corresponded to the mobility of BAK dimers (D), trimers (T) or higher molecular weight species. (TIF) [file pone.0049601.s002.tif]

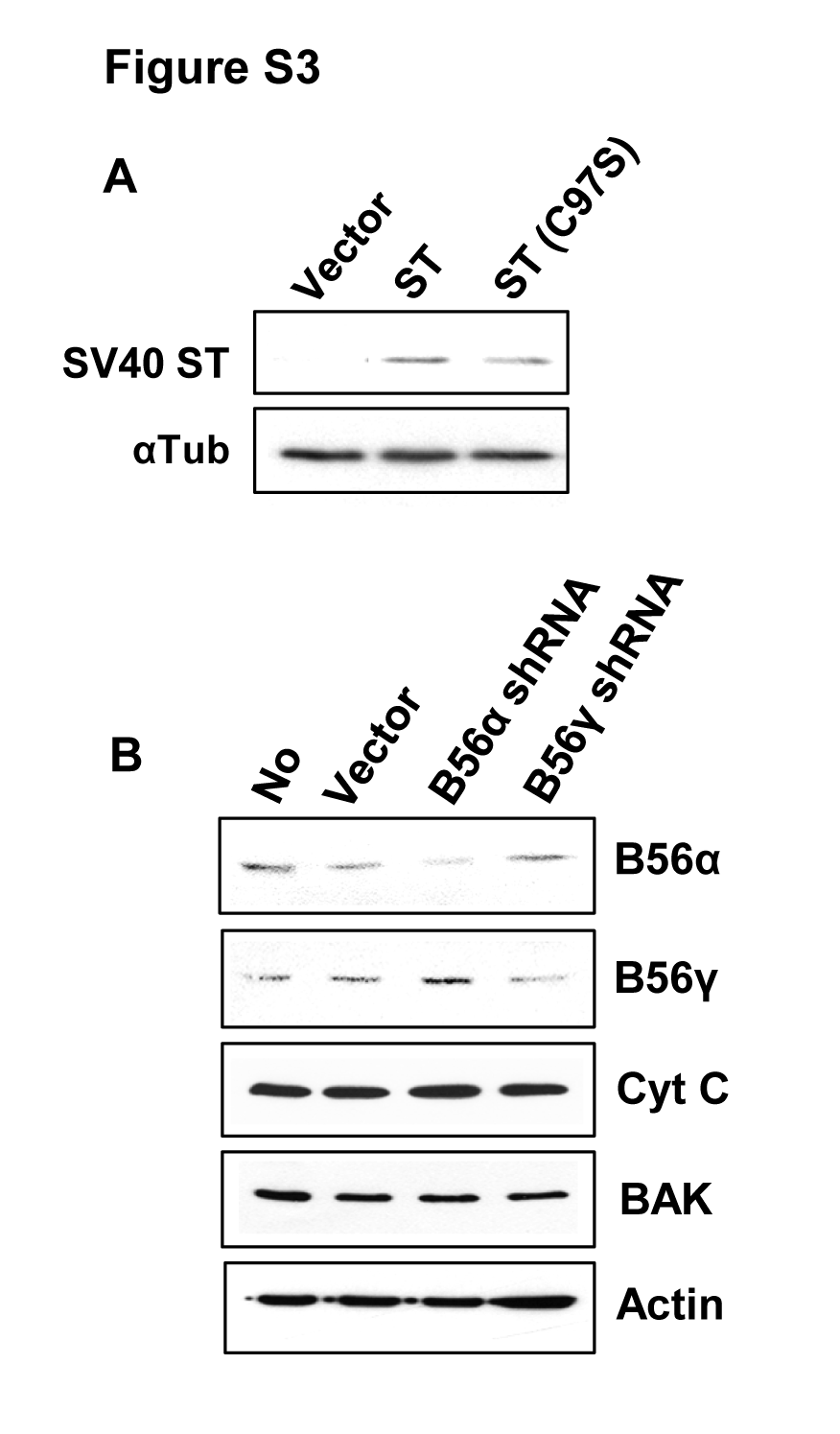

Supplement: Figure S3 — (A) HCT116-BAK cells were transfected with plasmids expressing either wild type SV40 ST or the C97S mutant that does not interact with or inhibit PP2A activity, or empty vector. Immunoblot analyses confirmed expression of either wild type or mutant SV40 ST using anti-ST monoclonal antibody (PAB280). α-Tubulin acted as loading control. (B) Silencing of PP2A-B56α and B56γ subunits by shRNA. Immunoblotting of lysates from cells transfected with either empty vector pMKO.1(EV) or pMKO.1 derivatives expressing specific shRNA for PP2A subunits B56α or B56γ. Remaining B56α and B56γ proteins were detected using specific antisera. Silencing of B56α and B56γ did not alter BAK or cytochrome c levels. Actin represents equal loading control. No = non-transfected cells, Vector = cells transfected with empty vector. (TIF) [file pone.0049601.s003.tif]

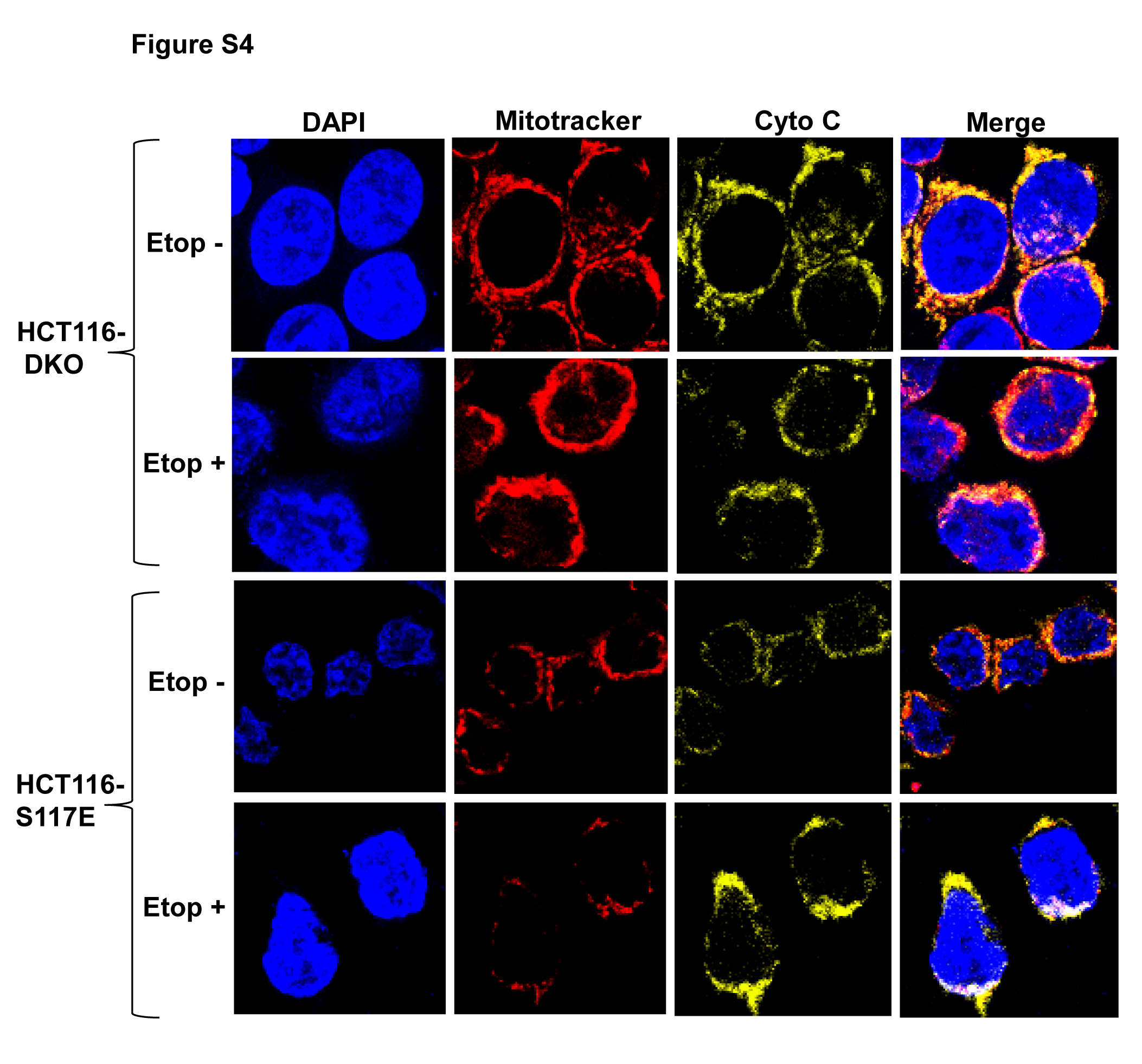

Supplement: Figure S4 — Confocal microscopy images of cytochrome c staining in cells treated ± etoposide. HCT116-DKO (double knockout bax−/−bak−/−) or HCT116-S117E mutant cells were fixed and stained for cytochrome c and analysed by confocal microscopy 24 hrs post treatment with etoposide. Mitotracker deep red FM was used to label mitochondria and nuclear DNA stained with DAPI. Both HCT116–DKO and S117E mutant failed to release cytochrome c after treated with etoposide. (TIF) [file pone.0049601.s004.tif]

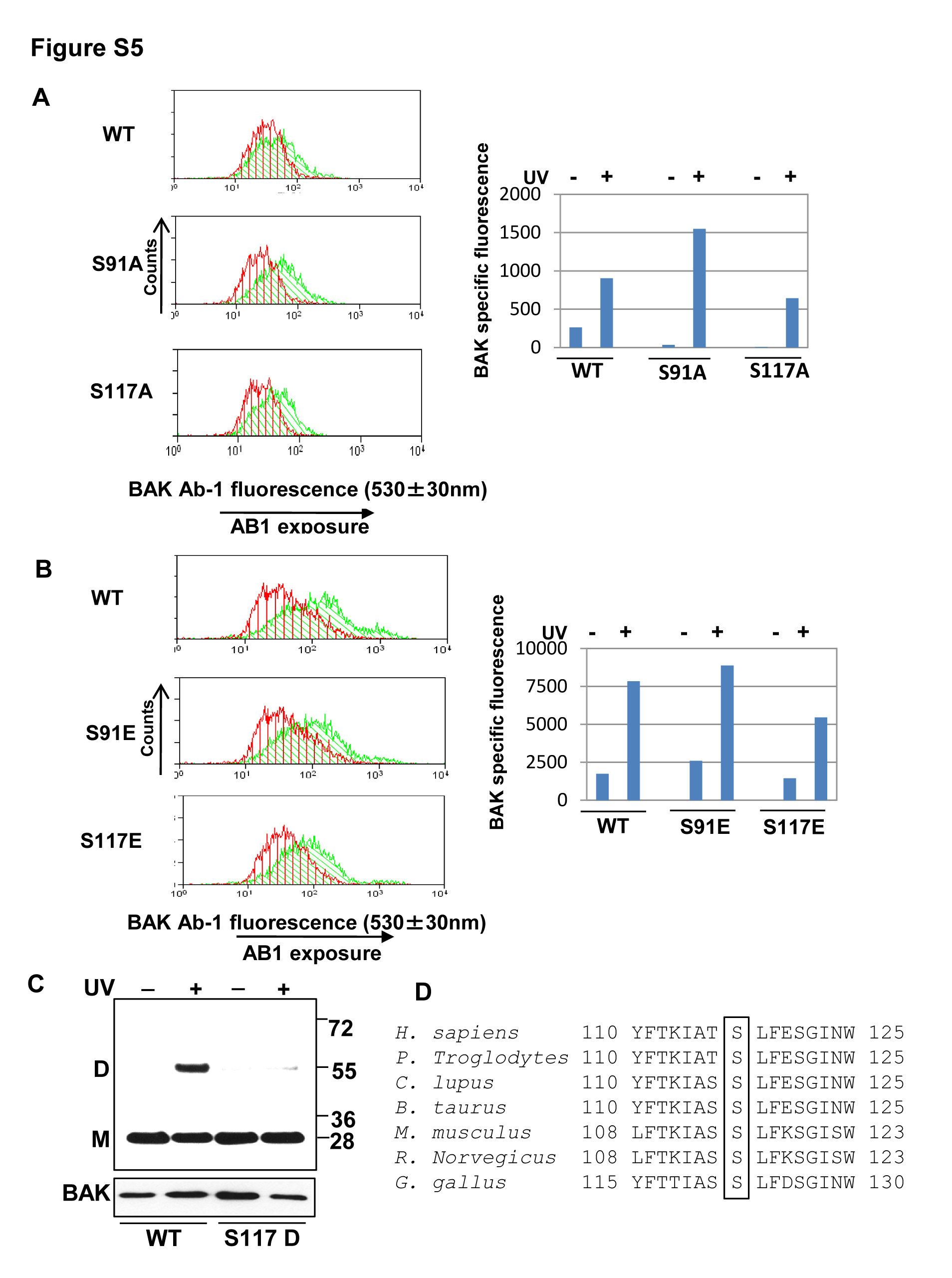

Supplement: Figure S5 — BAK mutants undergo N-terminal conformational change following DNA damage. (A) BAK conformational change was analysed in wild type (WT), S91A and S117A mutant cells ± DNA damage by UV (−, red, +, green) (left panel). Quantification of the BAK Ab1 specific fluorescence in WT, S91A and S117A (right panel). (B) BAK conformational change was also analysed in S91E and S117E mutants that also underwent N-terminal conformational change similar to wild type BAK (−, red, +, green) (left panel). Quantification of BAK-specific fluoresence (right panel). (C) BAK mutation S117D also impairs multimerization in response to UV damage using BMOE as cross-linking agent. (D) S117 is conserved amongst species. (TIF) [file pone.0049601.s005.tif]

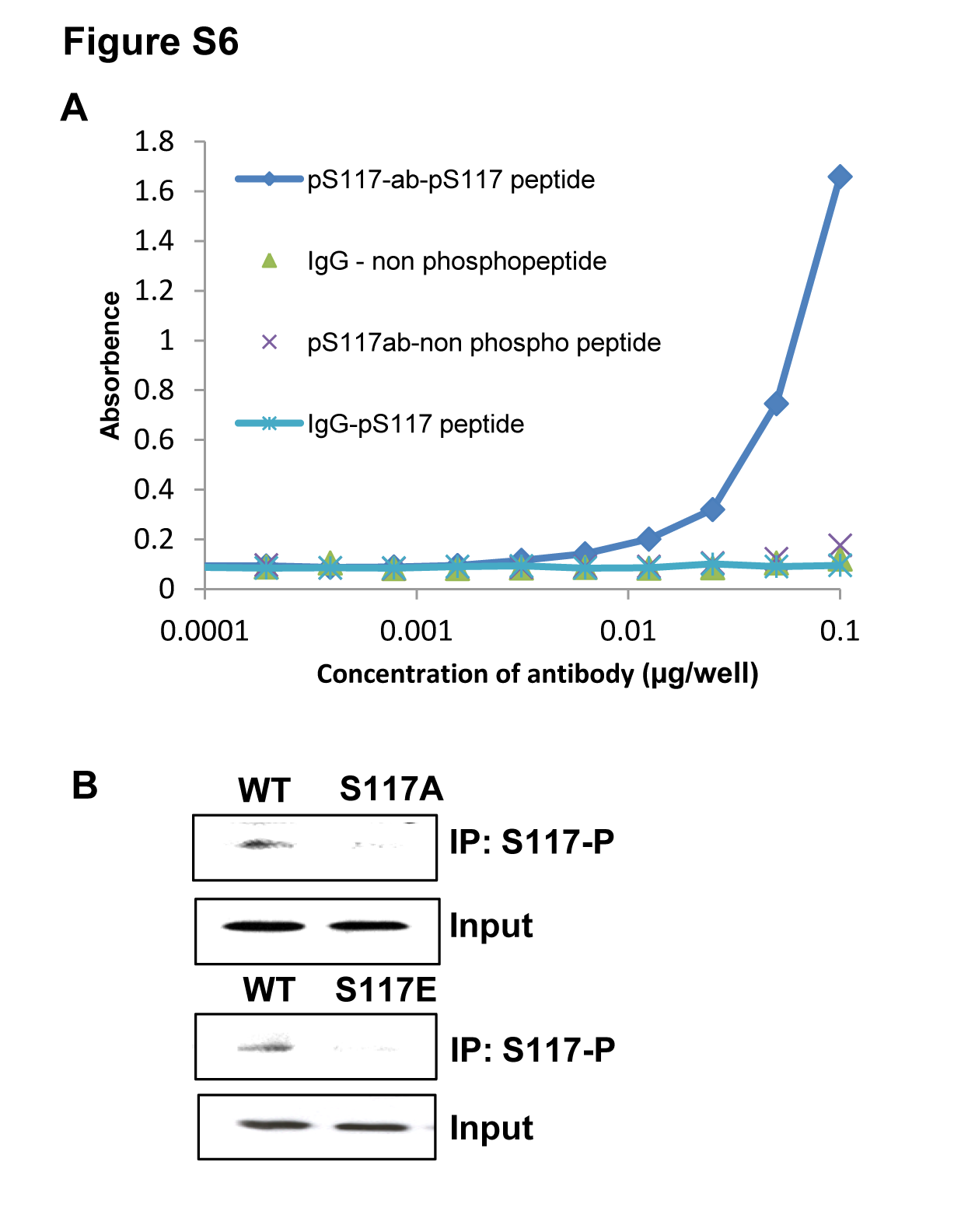

Supplement: Figure S6 — Characterization of anti-phospho S117 BAK polyclonal antiserum. (A) ELISA was performed with affinity purified polyclonal rabbit serum raised against a phospho S117 peptide. Reactivity was dependent upon the phospho group on the BAK peptide, rabbit IgG was used as a negative antibody control. (B) Specificity of phospho S117 BAK serum. Immunoprecipition (IP) of BAK was performed with phospho S117 serum using extracts from cells expressing either wild type BAK or the BAK S117A mutant (top panel), or wild type BAK or S117E mutant (bottom panel). Following IP, BAK was detected by western blotting with mouse anti-BAK Ab1. (TIF) [file pone.0049601.s006.tif]

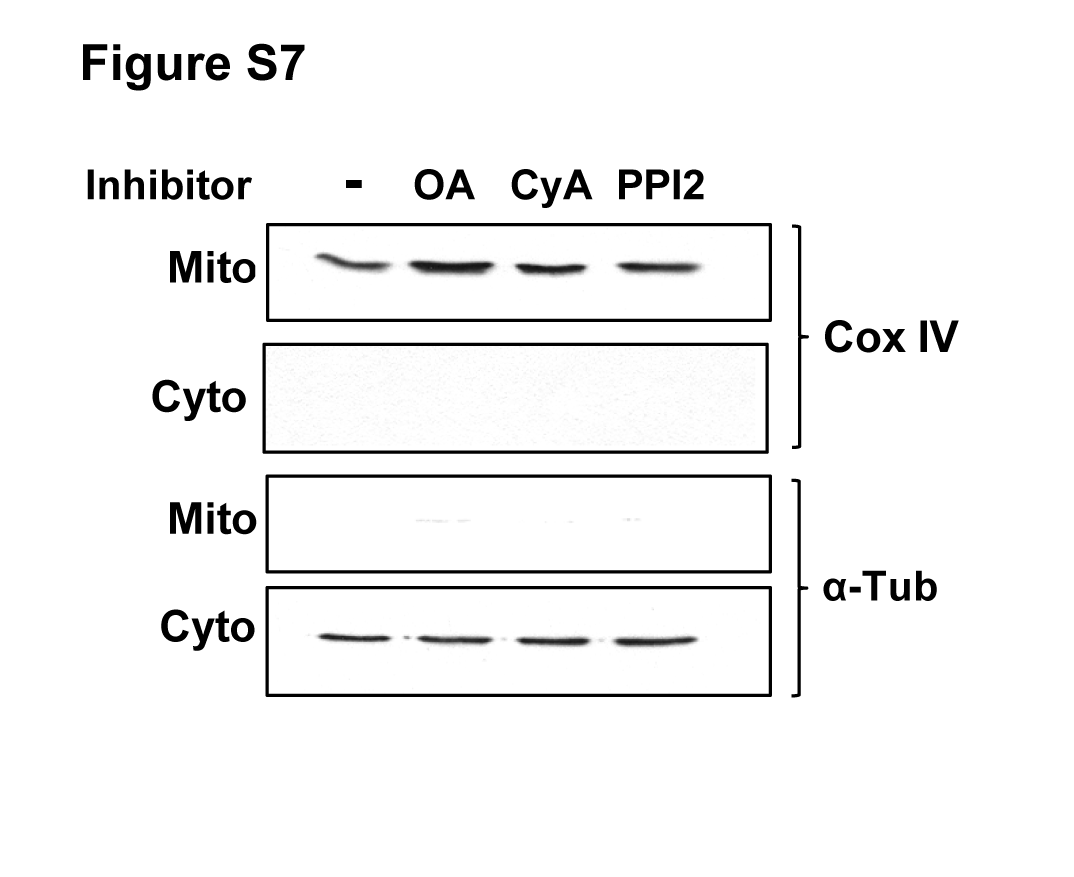

Supplement: Figure S7 — Purity of mitochondrial fractions. Fractionations were performed from HCT116-BAK expressing cells either treated ± with phosphatase inhibitors (OA, CyA and PPI 2) as indicated. After fractionation, mitochondrial and cytosolic extracts were assayed by western blot for cross-contamination using either Cox IV as a mitochondrial marker or tubulin as a cytosol specific protein. Mitochondria were then used for either multimerization, cytochrome c release or immunoprecipitation assays. (TIF) [file pone.0049601.s007.tif]

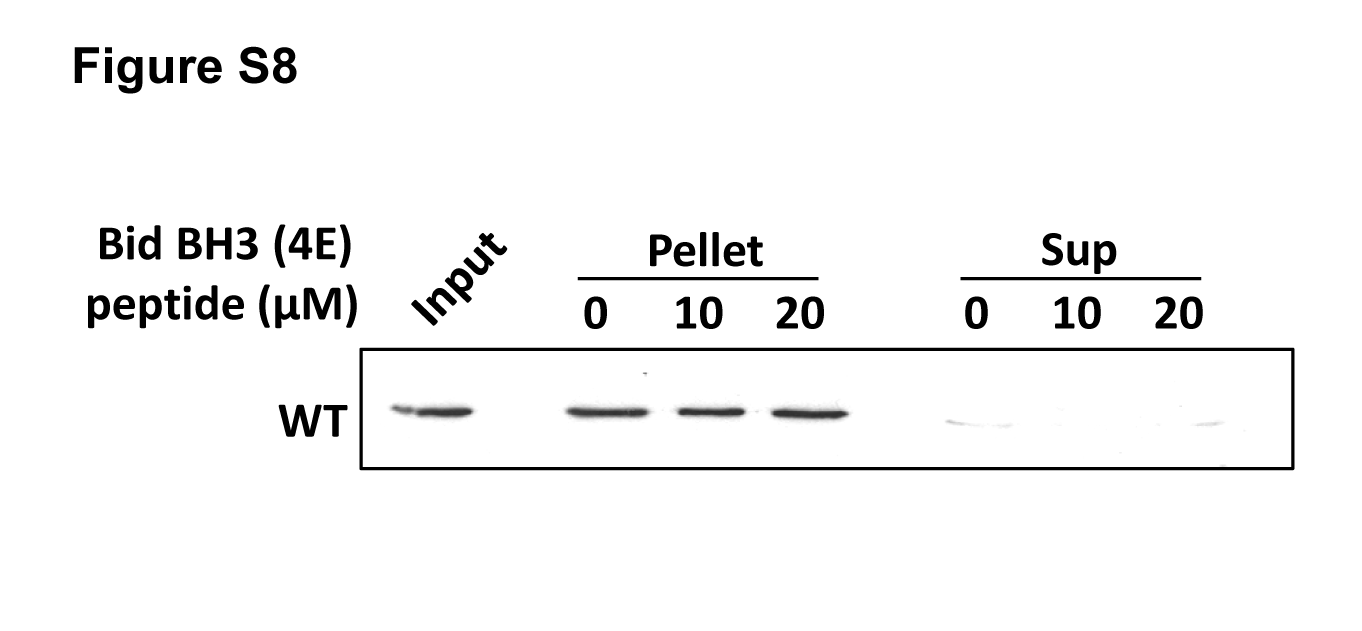

Supplement: Figure S8 — Mutant Bid BH3 peptide failed to release cytochrome c from HCT116-BAK expressing cells. Four conserved hydrophobic residues in the Bid BH3 peptide required for binding to BCL-2 family proteins were mutated to negatively charged glutamate (E) residues (BH3-4E). Cytochrome c release assays were performed with isolated mitochondria incubated with increasing concentrations of the mutant BH3 peptide. Following incubation with the peptide, mitochondria were harvested and cytochrome c retained in the mitochondria (pellet) or released into the supernatant (Sup) was analysed by immunoblot. (TIF) [file pone.0049601.s008.tif]

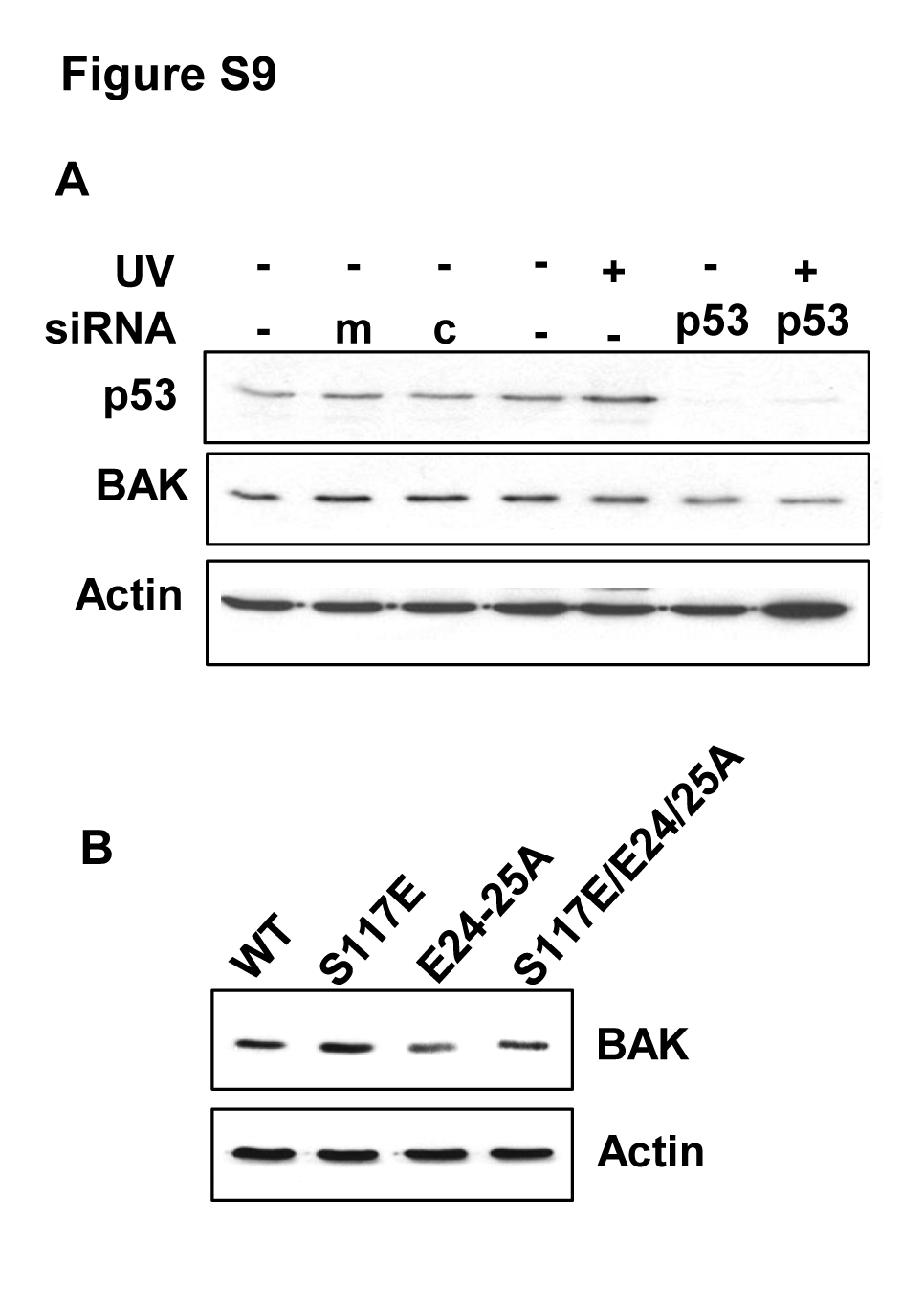

Supplement: Figure S9 — (A) siRNA silencing of p53 in HCT116-Bak cells was achieved with specific Oligos (Dharmacon). p53 protein levels were not recovered following UV damage in p53-silenced cells. BAK levels were unaffected by silencing of p53. Cells were also mock treated (m) with transfection reagent only, or transfected with control non-targeting siRNA (c). (B) Immunoblot of the BAK mutants S117E, the p53 binding site mutant E24-25A, or the triple mutant S117A/E24-25A were expressed to similar levels to the wild type protein in HCT116 cells. (TIF) [file pone.0049601.s009.tif]

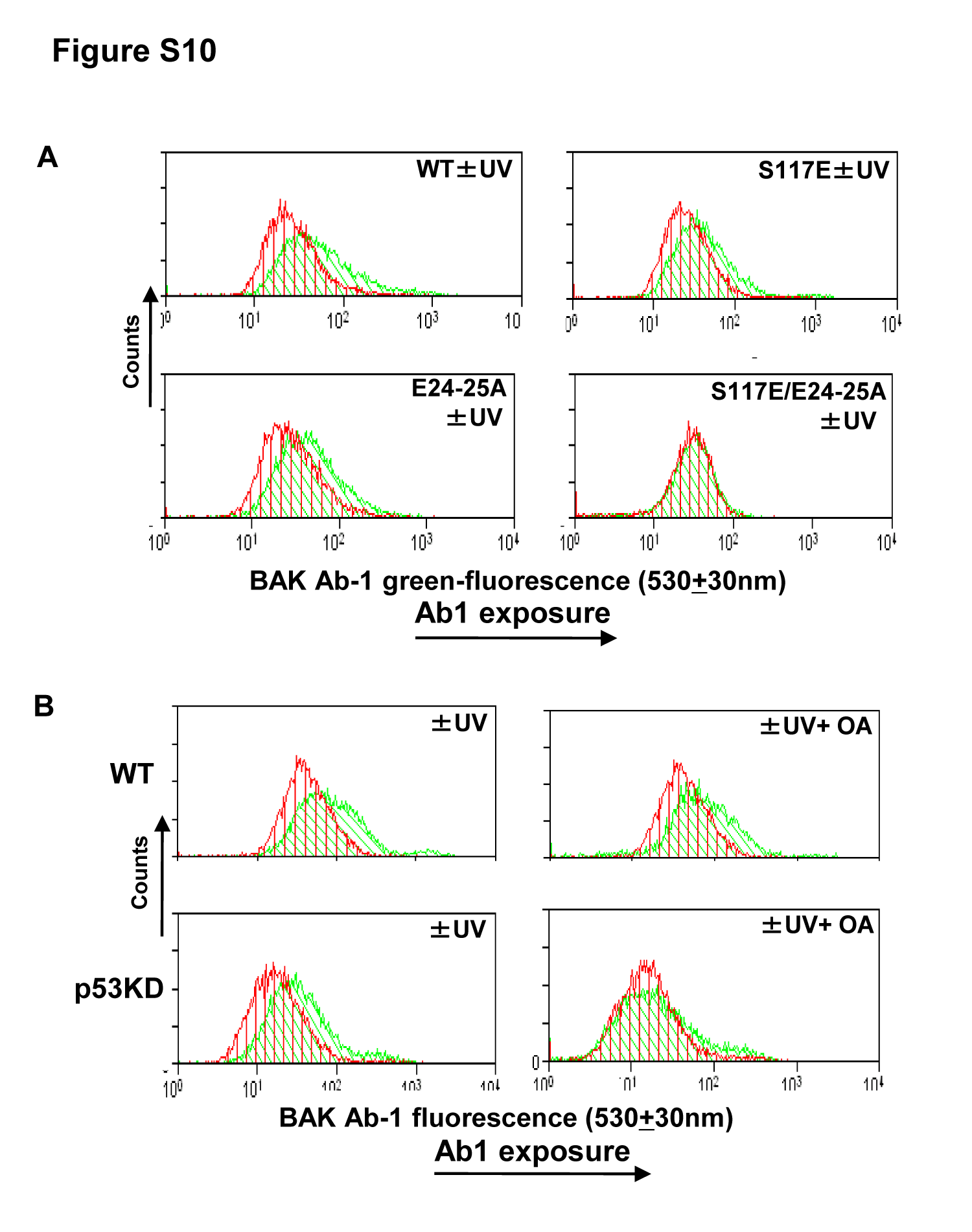

Supplement: Figure S10 — (A) FACS analyses of BAK N-terminal conformational change in HCT116-BAK wild type (WT), S117E, E24-25A or S117E/E24-25A cells following treatment ± with UV. The shift in fluoresence by binding of the BAK Ab-1 antibody following UV treatment is shown in green, compared to non-treated cells (red). (B) FACS analyses profile of HCT116-BAK wild type (WT) and cells in which p53 expression was knocked down by siRNA (p53KD) following exposure ± to UV and ± OA treatment. Red indicates no UV treatment, and green the profile from cells treated with UV. All experiments were performed at least 3 times, representative histograms are shown. (TIF) [file pone.0049601.s010.tif]
